# Rational design of a DNA sequence-specific modular protein tag by tuning the alkylation kinetics†
†Electronic supplementary information (ESI) available: Rate simulation of the cross-linking reaction (Fig. S1, S2), construction of the modular adaptor (Fig. S3, S15, and Table S15), evaluation of activity and orthogonality (Table S1–S12, Fig. S4–S11), structures of DNA origami scaffolds (Fig. S12), sequence of staple strand DNAs used to assemble the DNA origami scaffold (Table S13), and statistical analyses of AFM images for determining the occupancies of DNA scaffolds by modular adaptors (Fig. S13, S14, and Table S14). See DOI: 10.1039/c9sc02990g


**DOI:** 10.1039/c9sc02990g

**Published:** 2019-08-20

**Authors:** Thang Minh Nguyen, Eiji Nakata, Zhengxiao Zhang, Masayuki Saimura, Huyen Dinh, Takashi Morii

**Affiliations:** a Institute of Advanced Energy , Kyoto University , Uji , Kyoto 611-0011 , Japan . Email: t-morii@iae.kyoto-u.ac.jp

## Abstract

Sequence-selective chemical modification of DNA by synthetic ligands has been a long-standing challenge in the field of chemistry. Even when the ligand consists of a sequence-specific DNA binding domain and reactive group, sequence-selective reactions by these ligands are often accompanied by off-target reactions. A basic principle to design DNA modifiers that react at specific sites exclusively governed by DNA sequence recognition remains to be established. We have previously reported selective DNA modification by a self-ligating protein tag conjugated with a DNA-binding domain, termed as a modular adaptor, and orthogonal application of modular adaptors by relying on the chemoselectivity of the protein tag. The sequence-specific crosslinking reaction by the modular adaptor is thought to proceed in two steps: the first step involves the formation of a DNA–protein complex, while in the second step, a proximity-driven intermolecular crosslinking occurs. According to this scheme, the specific crosslinking reaction of a modular adaptor would be driven by the DNA recognition process only when the dissociation rate of the DNA complex is much higher than the rate constant for the alkylation reaction. In this study, as a proof of principle, a set of combinations for modular adaptors and their substrates were utilized to evaluate the reactions. Three types of modular adaptors consisting of a single type of self-ligating tag and three types of DNA binding proteins fulfill the kinetic requirements for the reaction of the self-ligating tag with a substrate and the dissociation of the DNA–protein complex. These modular adaptors actually undergo sequence-specific crosslinking reactions exclusively driven by the recognition of a specific DNA sequence. The design principle of sequence-specific modular adaptors based on the kinetic aspects of complex formation and chemical modification is applicable for developing recognition-driven selective modifiers for proteins and other biological macromolecules.

## Abstract 2

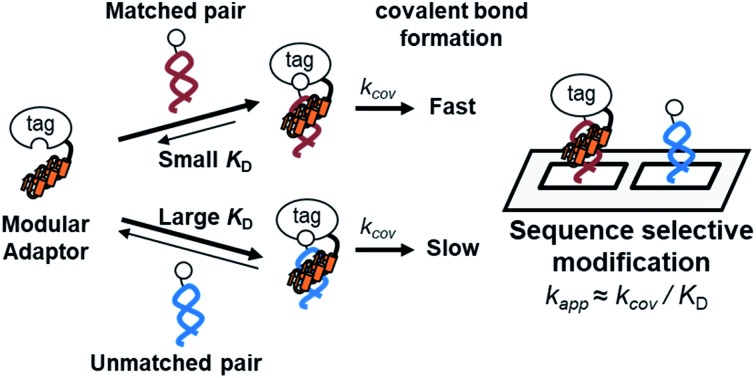
A design principle for sequence-specific DNA modifiers driven by the specific DNA recognition was proposed based on the kinetic parameters for DNA binding and modification reactions.

## Introduction

Site-specific chemical reactions that modify proteins and nucleic acids by ligands rely on specific molecular recognition of target sites and selective reactions.^1^ Sequence-selective chemical modification of DNA by DNA-binding molecules remains challenging in the field of chemistry.1d,e Various sequence-specific DNA binders, ranging from small molecules^2^ to short peptides,^3^ proteins,^4^ and synthetic oligonucleotides,^5^ have been coupled to a reactive group to specifically modify nucleic acid bases, sugar moieties, or phosphodiester linkages. However, coupling of a reactive group to a sequence-specific DNA binding domain is often accompanied by off-target reactions.^2^–^5^ A basic principle in designing DNA modifiers exclusively governed by DNA sequence recognition has not been established.

**Fig. 1:**
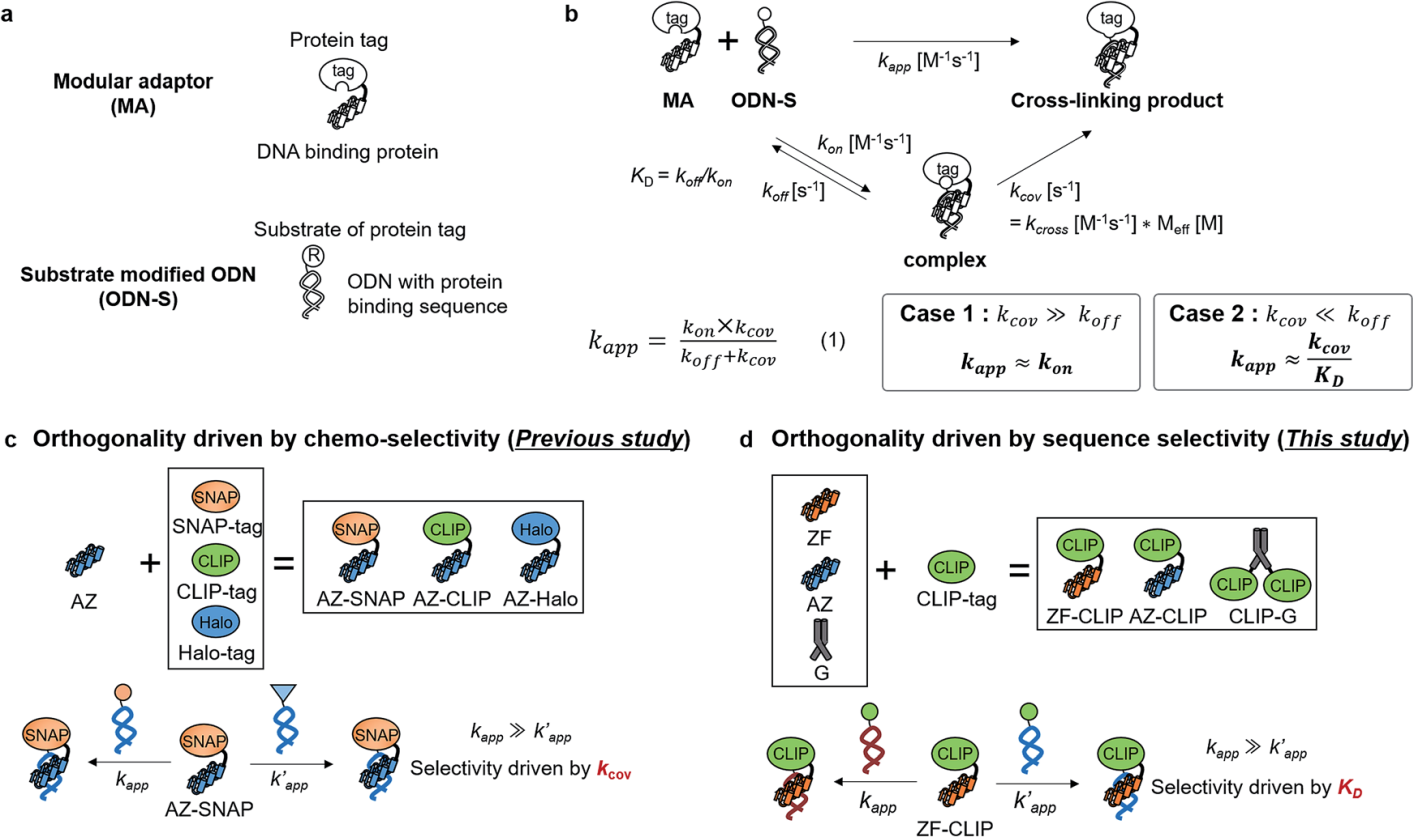
(a) Structures and elements of the modular adaptor (MA) and substrate-modified oligodeoxyribonucleotide (ODN-S). (b) Scheme of the cross-linking reaction between MA and ODN-S. Complex formation between MA and ODN-S is governed by the equilibrium dissociation constant (*K*_D_) [M] as defined by *K*_D_ = *k*_off_/*k*_on_. The apparent rate constant (*k*_app_) [M^–1^ s^–1^] is expressed as *k*_on_ and *k*_off_ (or *K*_D_) and the rate constant *k*_cov_ [s^–1^] for the proximity-driven intermolecular crosslinking reaction between MA and ODN-S is defined by the rate constant of intermolecular covalent formation (*k*_cross_) [M^–1^ s^–1^] and the effective concentration (*M*_eff_) [M] (eqn (1)). (c) Orthogonal cross-linking reactions driven by the chemo-selectivity of the protein tag described in our previous study.^15^ The apparent reaction rate constants for the matched pair and the unmatched pair are indicated as *k*_app_ [M^–1^ s^–1^] and 
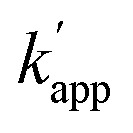
 [M^–1^ s^–1^], respectively. The selective cross-linking reaction proceeds when 
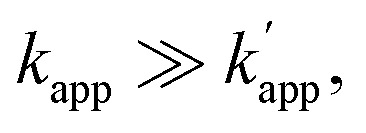
 which is realized only when *k*_cov_ for the matched pair of the substrate of protein tags is much larger than that for the unmatched pair of the substrate of protein tags. (d) Orthogonal cross-linking reactions driven by the sequence selectivity of DNA-binding domains and chemo-selective cross-linking reactions by the protein tag described in this study. The selective reactions of ZF-CLIP, AZ-CLIP, and CLIP-G proceed with *k*_app_ in case 2 of (b) with selectivity mainly governed by *K*_D_.

Fully addressable nano-architectures of various shapes and geometries provide ideal scaffolds for locating molecules of interest at defined positions with a nanometer spatial precision.^6^ DNA scaffolds prepared by DNA origami^7^ can be readily incorporated with additional DNA sequences on their surface by simple extension of the staple strand. These extended DNA strands are further chemically modified to serve as binding sites for various molecules.^8^ Proteins are a particularly interesting class of molecules for assembly on the DNA scaffold because of their wide functional variability.^9^ For assembly on the DNA scaffold through hybridization, a protein of interest (POI) is chemically modified with a short DNA strand that is complementary to the attached sequence on the DNA scaffold.^10^ Although this method is convenient, it has some limitations; for example, the modification conditions are limited and may cause loss of activity.^11^,^12^ It would be ideal to locate the POI at a specific position on the DNA scaffold without the requirement for post-chemical modification of the POI to prepare a precisely controlled assembly suitable for further investigation.9c,^13^ To achieve efficient and rapid placement of a POI at a specific DNA site, we developed modular adaptors (Fig. 1a and b).^14^,^15^ A modular adaptor consists of a DNA-binding protein (zif268^16^) that functions as a DNA sequence-selective recognition module and self-ligating protein tag (SNAP-tag^17^) which forms a chemoselective covalent linkage between the protein tag and its substrate *O*^2^-benzylguanine (BG) modified at the given DNA sequence (Fig. 1a).^14^ A modular adaptor-fused enzyme was specifically located at the target position of the DNA scaffold with fast reaction kinetics and in high yield while fully retaining the original enzymatic activity.^18^ In order to assemble three types of proteins at unique positions on the DNA scaffold, a set of modular adaptors with orthogonality and which retained fast reaction kinetics under mild conditions was selected from the systematic combination of DNA-binding domains (zif268 or AZP4 19) and protein tags, SNAP-tag, CLIP-tag,^20^ and Halo-tag.^15^,^21^ Among them, three types of orthogonal modular adaptors simultaneously directed the three types of proteins to their respective positions on the DNA scaffold. In a previous study, orthogonal cross-linking reactions of modular adaptors were mainly conducted by taking advantage of the chemoselective characteristics of self-ligating protein tags (Fig. 1c), not exclusively by the sequence selectivity of the DNA-binding protein. In fact, zif268 conjugated with SNAP-tag (ZF-SNAP) and AZP4 conjugated with SNAP-tag (AZ-SNAP) reacted to the same extent with BG at both the zif268 and AZP4 binding sequences.^15^ This non-sequence-selective modification was discussed by considering the kinetics of the sequence-specific crosslinking reaction by the modular adaptor in two steps: the first step is the formation of a DNA–protein complex, and a proximity-driven intermolecular crosslinking reaction is initiated in the second step (Fig. 1b). According to this scheme, the specific crosslinking reaction of a modular adaptor would be driven by the DNA recognition process only when the dissociation rate of the DNA complex is much higher than the rate constant for the alkylation reaction.

**Fig. 2:**
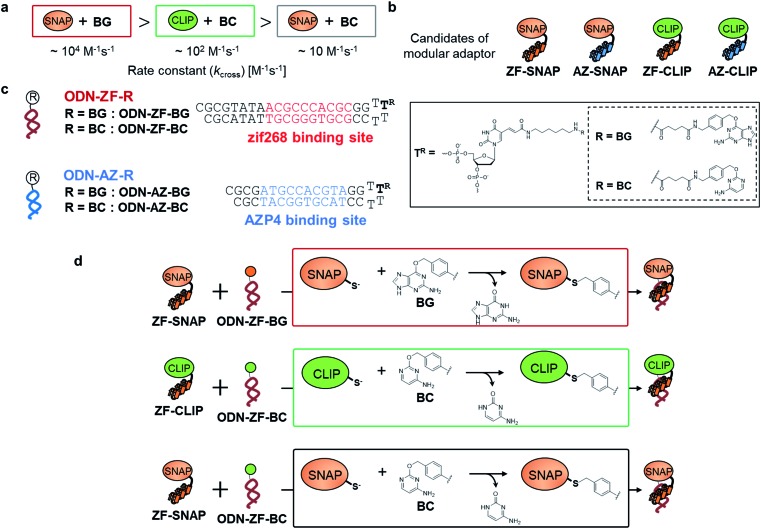
(a) Comparison of the reactivity of the combination of the protein tag and substrate (*k*_cross_). (b) Candidates for the modular adaptor used in this study. (c) Nucleotide sequences of ODNs for each modular adaptor with the substrate for protein tags and chemical structures of the substrates for protein tag-modified T, denoted as “T^R^”. (d) Reaction schemes representing the cross-linking reactions between the modular adaptors (ZF-SNAP or ZF-CLIP) and substrates incorporated into ODN-S (ODN-ZF-BG and ODN-ZF-BC).

In this study, as a proof of principle for the above concept, we constructed sequence-selective modular adaptors by tuning the reaction kinetics between the self-ligating protein tag and its substrate (Fig. 2a and d). Conjugation of different types of DNA-binding domains to the same self-ligating protein tag provides a variety of orthogonal modular adaptors, in which the crosslinking reaction is exclusively governed by sequence-specific DNA recognition (Fig. 1d).

The reaction of the modular adaptor with its substrate at the specific DNA sequence proceeds through specific recognition of the DNA sequence and an alkylation reaction between the protein tag and its substrate (Fig. 1b). Therefore, the factors governing the specific reactions of modular adaptors are well-correlated with the design of selective DNA modifiers driven by sequence-specific DNA recognition.^18^,^22^ Our design principle of modular adaptors based on the kinetic parameters of the DNA binding and alkylation reaction would be applicable to design a wide range of site-specific modifiers driven by target recognition.

## Results

### Kinetic aspects of the modular adaptor provide a design strategy for sequence-specific modular adaptors for orthogonal covalent bond formation with the substrate-modified ODNs

Our previous study of the reaction of modular adaptors, consisting of a DNA-binding domain and a cross-link forming protein tag (Fig. 1a), suggested important factors that affected the kinetics and selectivity of the cross-linking reaction. Covalent bond formation between the modular adaptor and its substrate-modified oligodeoxyribonucleotide (ODN-S) (Fig. 1a) occurs in the following two steps (Fig. 1b): a reversible complex forms between the DNA-binding domain and ODN-S, and then a proximity-driven intermolecular cross-linking reaction occurs between the self-ligating protein tag domain and its substrate on ODN-S.^15^ The apparent rate constant of the cross-linking reaction (*k*_app_) (M^–1^ s^–1^) between the modular adaptor and substrate-modified DNA is represented by eqn (1) shown in Fig. 1b. In eqn (1), a modular adaptor with a highly reactive protein tag to its substrate (*k*_cov_ ≫ *k*_off_) falls into case 1.^23^,^24^ Because the association rates (*k*_on_) of DNA-binding proteins to their specific and non-specific sites are nearly identical,^24^ the modular adaptor is thought to react with ODN-S regardless of the DNA sequence (case 1 in Fig. 1b). Therefore, reducing the reactivity of the protein tag with the substrate (*k*_cov_) to fit into the condition of case 2, *k*_cov_ ≪ *k*_off_ (Fig. 1b), would be an effective strategy for preventing the cross-linking reaction at unmatched DNA sequences by modular adaptors. In case 2, a higher equilibrium dissociation constant (*K*_D_) for the unmatched complex of the zinc finger motif and ODNs results in fewer cross-linking reactions of unmatched complexes. The difference in the cross-linking reactions for the matched and unmatched pairs evaluated in our previous study^15^ and this study is described in Fig. 1c and d, respectively.

Time-dependent changes in the yield of the cross-linking reaction (Fig. S1†) were simulated by varying the apparent rate constants. In addition, the apparent reaction rate constant of the matched pair (*k*_app_) and that of the unmatched pair 
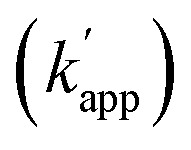
 were investigated with various *k*_cov_ values (Fig. S2†). From these simulations, the sequence-selective orthogonal cross-linking reaction takes place when the rate constant of the matched pair (*k*_app_) is two orders of magnitude higher than that of the unmatched pair 
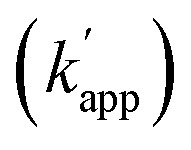
. Thus, to achieve the sequence-selective crosslinking reaction, the reactivity of the cross-linking reaction should be finely tuned to meet the condition of case 2.

**Fig. 3:**
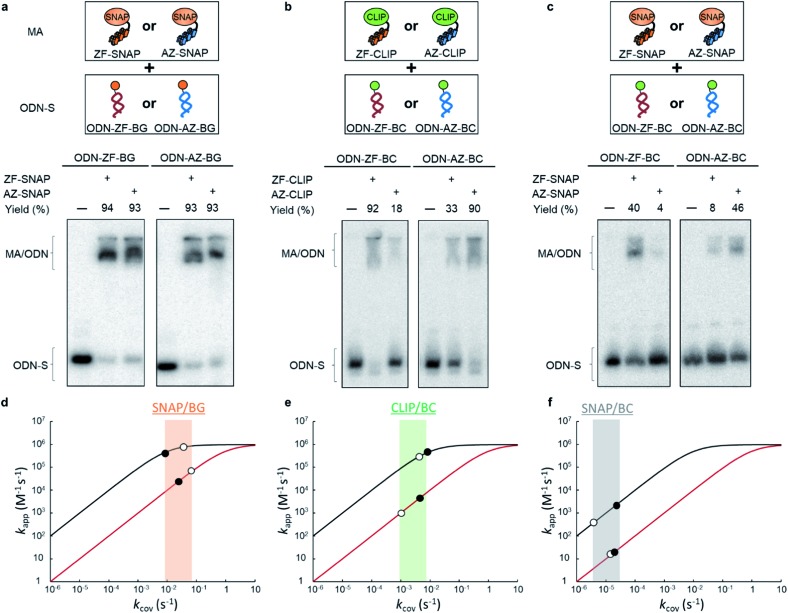
Analyses of cross-linking reactions between the modular adaptors and the substrate-modified ODNs. (a–c) Denaturing PAGE analyses of the cross-linking reaction by modular adaptors (MA: ZF-SNAP, AZ-SNAP, ZF-CLIP and AZ-CLIP) and each ODN-S (ODN-ZF-BG, ODN-AZ-BG, ODN-ZF-BC and ODN-AZ-BC). Each 5′-^32^P-end-labeled ODN-S was incubated with a modular adaptor (100 nM) for 30 min in a buffer (pH 8.0) containing 40 mM Tris–HCl, 20 mM acetic acid, 12.5 mM MgCl_2_, 1 mM DTT, 1 μM ZnCl_2_, 0.02% Tween 20, 200 nM BSA and 100 nM calf thymus DNA at ambient temperature. (d–f) Simulation of *k*_app_ with various *k*_cov_ by following eqn (1) (Fig. 1b). The *k*_app_ values for the matched and unmatched pairs of MA and ODN-S at given *k*_cov_ are shown in black and red curves, respectively. The details of simulation conditions are shown in Fig. S2.† The experimentally obtained *k*_app_ values (Table 1) are marked on the simulated lines. Filled and open circles indicate MA conjugated with ZF and AZ, respectively. (d) Reactions of SNAP-tag conjugated MAs with BG. (e) Reactions of CLIP-tag conjugated MAs with BC. (f) Reactions of SNAP-tag conjugated MAs with BC.

To test the above hypothesis, three combinations of protein tags and substrates, SNAP-tag with BG (∼10^4^ M^–1^ s^–1^),^17^,^20^ CLIP-tag with *O*^2^-benzylcytosine (BC) (∼10^2^ M^–1^ s^–1^),^20^ and SNAP-tag with BC (∼10 M^–1^ s^–1^),^20^ were prepared for the cross-link-forming domain of the modular adaptor (Fig. 2a). Two DNA binding proteins, zif268 (ZF) and AZP4 (AZ), were utilized as the DNA-binding domain of the modular adaptor (Fig. 2b). ZF-CLIP was constructed by fusing CLIP-tag to the C-terminus of zif268 through a GGSGGS linker (Fig. S3a†). ZF-SNAP, AZ-SNAP, and AZ-CLIP were prepared as described previously.^15^ ODNs containing the zif268- or AZP4-binding sequence were designed to form a loop with four T nucleotides, in which one of the T nucleotides was replaced with amino-C6-T (ODN-ZF or ODN-AZ). Amino-C6-T was modified with each of the substrates, BG or BC (ODN-ZF-BG, ODN-ZF-BC, ODN-AZ-BG or ODN-AZ-BC, respectively, as shown in Fig. 2c). Complex formations by ZF or AZ derivatives and ODN-ZF or ODN-AZ were titrated by means of fluorescence polarization measurements to determine the *K*_D_ values (Fig. S4 and Table S1, S2†). The results confirmed that the *K*_D_ values for the matched and unmatched complexes differed by more than two orders of magnitude. Cross-linking reactions of these modular adaptors with ODN-S containing the respective matched or unmatched sequence were analysed by denaturing polyacrylamide gel electrophoresis (PAGE) as shown in Fig. 3. In the combination of SNAP-tag and BG, *k*_app_ values for the reactions of the matched pair ZF-SNAP with ODN-ZF-BG and AZ-SNAP with ODN-AZ-BG were 3.8 × 10^5^ and 9.1 × 10^5^ M^–1^ s^–1^, respectively (Table 1). For the combination of CLIP-tag and BC, *k*_app_ values for the reactions of the matched pair ZF-CLIP with ODN-ZF-BC and AZ-CLIP with ODN-AZ-BC were 7.1 × 10^5^ and 5.0 × 10^5^ M^–1^ s^–1^, respectively (Table 1). In the combination of SNAP-tag with BC, *k*_app_ values for the reactions of the matched pair ZF-SNAP with ODN-ZF-BC and AZ-SNAP with ODN-AZ-BC were 1.7 × 10^3^ and 3.0 × 10^2^ M^–1^ s^–1^, respectively (Table 1). The cross-linking reactions between ZF-SNAP and ODN-ZF-BG, AZ-SNAP and ODN-AZ-BG, ZF-CLIP and ODN-ZF-BC, and AZ-CLIP and ODN-AZ-BC gave yields of 94%, 93%, 92%, and 90%, respectively (Fig. 3a and b). The reactions of ZF-SNAP with ODN-ZF-BC and AZ-SNAP with ODN-AZ-BC were very slow and showed yields of 40% and 46%, respectively, after 30 min of incubation under the described conditions (Fig. 3c).

**Table 1:** Kinetic parameters 
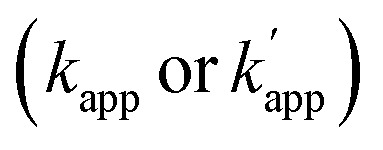
 for the cross-linking reactions between ODN-S and MAs

MAs	*k*_app_ (M^–1^ s^–1^)	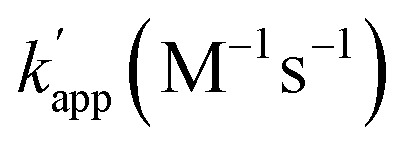	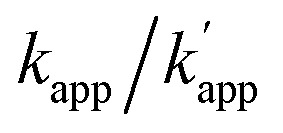
	(ODN-S)	(ODN-S)	
ZF-SNAP	(3.8 ± 1.2) × 10^5^	(2.6 ± 0.2) × 10^4^	15
	(ODN-ZF-BG)	(ODN-AZ-BG)	
AZ-SNAP	(9.1 ± 2.4) × 10^5^	(9.5 ± 0.6) × 10^4^	10
	(ODN-AZ-BG)	(ODN-ZF-BG)	
ZF-CLIP	(7.1 ± 0.2) × 10^5^	(2.8 ± 0.2) × 10^3^	254
	(ODN-ZF-BC)	(ODN-AZ-BC)	
AZ-CLIP	(5.0 ± 0.7) × 10^5^	(8.1 ± 2.7) × 10^2^	617
	(ODN-AZ-BC)	(ODN-ZF-BC)	
ZF-SNAP	(1.7 ± 0.2) × 10^3^	22 ± 1	77
	(ODN-ZF-BC)	(ODN-AZ-BC)	
AZ-SNAP	(3.0 ± 0.1) × 10^2^	11 ± 3	27
	(ODN-AZ-BC)	(ODN-ZF-BC)	

Next, we evaluated the reactions of unmatched pairs for each combination of the protein tag and substrate (Table 1). The 
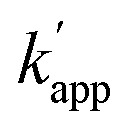
 values of the unmatched pairs of ZF-SNAP with ODN-AZ-BG and AZ-SNAP with ODN-ZF-BG were 2.6 × 10^4^ and 9.5 × 10^4^ M^–1^ s^–1^, respectively. The 
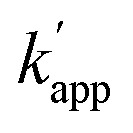
 values of the unmatched pairs of ZF-CLIP with ODN-AZ-BC and AZ-CLIP with ODN-ZF-BC were 2.8 × 10^3^ and 8.1 × 10^2^ M^–1^ s^–1^, respectively. The 
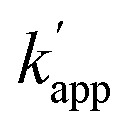
 values of the unmatched pairs of ZF-SNAP with ODN-AZ-BC and AZ-SNAP with ODN-ZF-BC were 22 and 11 M^–1^ s^–1^, respectively (Table 1). The 
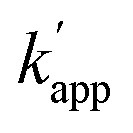
 values of unmatched pairs of ZF-SNAP and AZ-SNAP were one to two orders of magnitude lower than the *k*_app_ of the matched pairs. Thus, the sequence-specific reaction only minimally occurred, as shown in Fig. S1.† In contrast, the 
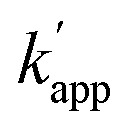
 values of ZF-CLIP and AZ-CLIP were two orders of magnitude lower than *k*_app_ of the matched pairs (Fig. S5† and Table 1). In the combination of CLIP-tag and BC, the observed differences in the apparent second-order rate constants were parallel to the difference in the equilibrium dissociation constants for the matched and unmatched complexes of ZF-CLIP and AZ-CLIP, as expected from eqn (1) in case 2 (Fig. 1b). These results indicated that the cross-linking reaction of the modular adaptors consisting of CLIP-tag with the substrate BC on ODNs proceeded as shown in case 2 of eqn (1). Because the larger *k*_app_ for the matched pair was preferred as long as it falls within case 2, the pair of CLIP-tag and BC was selected as the self-ligating protein tag domain for modular adaptors that would form a covalent linkage at a specific sequence driven by the DNA recognition domain.

### Sequence-specific reactions of modular adaptors consisting of CLIP-tag with BC modified ODNs

**Fig. 4:**
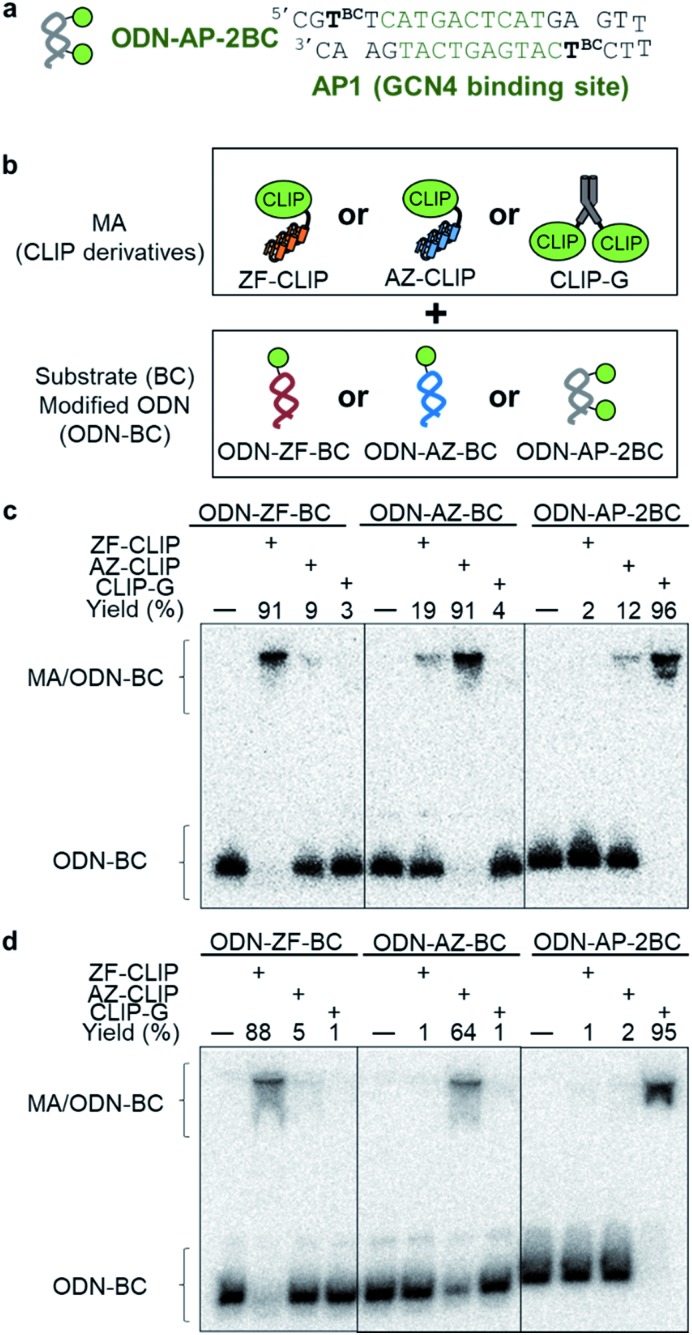
(a) Nucleotide sequences of ODNs for GCN4 with BC modified T, denoted as “T^BC^” (ODN-AP-2BC). (b) Illustrations of the optimized sequence-specific modular adaptors (MAs) and substrate-modified ODN (ODN-S). (c and d) Autoradiograms show denaturing PAGE analyses of the crosslinking reaction of MA (ZF-CLIP, AZ-CLIP, and CLIP-G) and ODN-S (ODN-ZF-BC, ODN-AZ-BC, and ODN-AP-2BC) with (c) or without 200 mM NaCl (d). Each 5′-^32^P-end-labeled ODN-S (0.5 nM) was incubated with 50 nM each of MAs (c) or 200 nM ZF-CLIP, 500 nM AZ-CLIP, or 100 nM CLIP-G (d), respectively, for 10 min (c) and 60 min (d). These reactions were carried out in a buffer (pH 8.0) containing 40 mM Tris–HCl, 20 mM acetic acid, 12.5 mM MgCl_2_, 1 mM DTT, 1 μM ZnCl_2_, and 200 mM NaCl in (d), and 0.02% Tween 20 at ambient temperature.

Three types of modular adaptors bearing the same self-ligating protein tag but different DNA-binding domains (ZF-CLIP, AZ-CLIP, and CLIP-G) were tested in a sequence-selective crosslinking reaction with ODNs modified with BC (Fig. 4). CLIP-G consisted of a basic-leucine zipper (bZIP) class of protein GCN4 25 as a homo-dimeric DNA binding module and CLIP-tag fused to the N-terminus of GCN4 through a GGSGGS linker. An ODN containing the AP1 sequence as the GCN4 binding site was designed for the matched target for CLIP-G, in which two T nucleotides at either ends of the AP1 sequence were displaced by amino-C6-T (ODN-AP). ODN-AP was further modified with BC (ODN-AP-2BC) as the substrate of CLIP-G (Fig. S3b and c†). The cross-linking reactions of ZF-CLIP, AZ-CLIP, and CLIP-G with their respective matched ODNs were nearly orthogonal, as shown in Fig. 4c, with more than 90% yield for matched pairs and less than 19% covalent bond formation for unmatched pairs. The observed sequence-specific cross-linking reactions of ZF-CLIP, AZ-CLIP, and CLIP-G were driven by the specific DNA binding of each modular adaptor (Fig. S5, Table S3†). The *k*_app_ for the reaction of CLIP-G and its matched sequence ODN-AP-2BC was determined to be 2.1 × 10^6^ M^–1^ s^–1^ and 
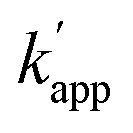
 values ranged from 10^3^ to 10^4^ M^–1^ s^–1^. For the reactions of unmatched pairs, ZF-CLIP or AZ-CLIP with ODN-AP-2BC, the 
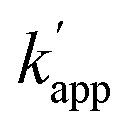
 values were 10^2^–10^3^ M^–1^ s^–1^. Furthermore, in the presence of 200 mM NaCl, non-specific DNA binding was reduced (Fig. S6 and Table S4†)^26^,^27^ and covalent bond formation at the unmatched pairs were further reduced to less than 5% (Fig. 4d). The yields of the matched pairs were also reduced for AZ-CLIP (64%) and ZF-CLIP (88%). The kinetic constants of the matched and unmatched pairs for SNAP-tag and CLIP-tag in the presence of 200 mM NaCl are summarized in Fig. S7 and Tables S5 and S6,† respectively. Under this condition, the sequence-specificity of ZF-CLIP to the consensus sequence of zif268 (GCGTGGGCGT) and its mutated sequences was further investigated (Table S7†). ODN-ZF(G/T) and ODN-ZF(G/C) contained a single nucleotide substitution in the consensus sequence and ODN-ZF(GG/TC) and ODN-ZF(GC/CT) were mutated with two nucleotides. The *K*_D_ values reported for the complexes of zif268 and these sequences were more than one order of magnitude higher than those with the consensus sequence.^24^ Actually, the *K*_D_ values determined for the binding complexes of ZF-CLIP and these mutated ODNs by means of a fluorescence polarization assay were more than one order of magnitude higher for single nucleotide substituted ODN-ZF(G/T) and ODN-ZF(G/C) and two orders of magnitude higher for two nucleotide substituted ODN-ZF(GG/TC) and ODN-ZF(GC/CT) than that for ODN-ZF (Fig. S8 and Table S8†), being consistent with previous studies.^24^ The *k*_app_ for the reaction of ZF-CLIP and ODN-ZF-BC in the presence of 200 mM NaCl was determined to be 5.1 × 10^4^ M^–1^ s^–1^. For the reaction with these mutated ODNs, the 
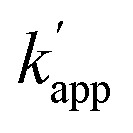
 values for the single nucleotide substituted ODN-ZF(G/T) and ODN-ZF(G/C) were 1.6 × 10^3^ and 2.3 × 10^3^ M^–1^ s^–1^, respectively (Fig. S9 and Table S9†). The 
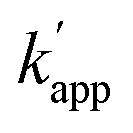
 for the two nucleotide substituted ODNs ODN-ZF(GG/TC) and ODN-ZF(GC/CT) could not be obtained because of their very slow reactions. These results clearly demonstrated that the sequence selective cross-linking reaction of the modular adaptor consisting of CLIP-tag with the substrate BC on ODNs proceeded by discriminating single nucleotide substitution, and indicated that the selectivity of the crosslinking reaction was governed by the *K*_D_ of the binding complex.

### Coassembly of modular adaptors on a DNA scaffold

**Fig. 5:**
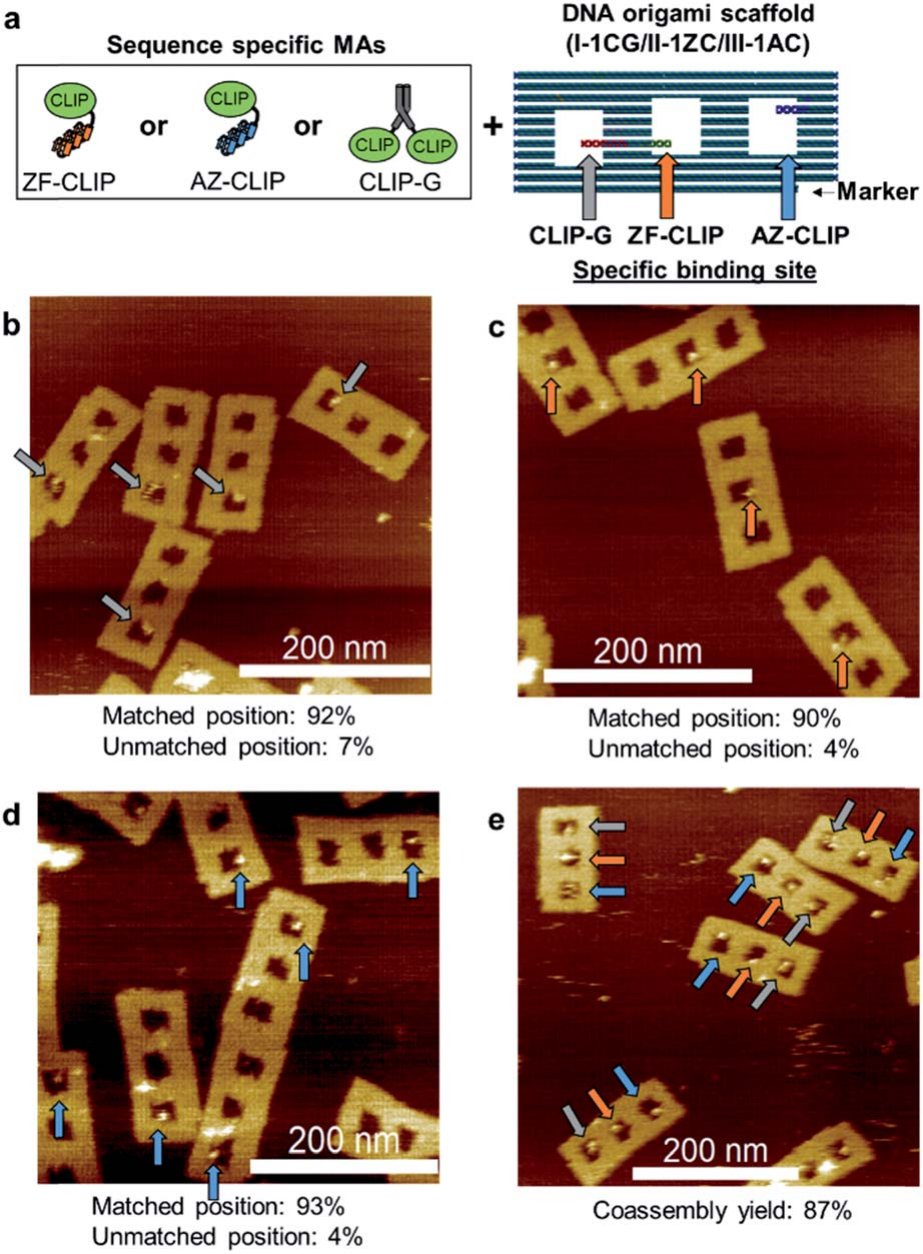
Three types of sequence-specific MAs orthogonally reacted at the predesigned positions on the DNA scaffold. (a) Three types of MAs and the DNA origami scaffold (I-1CG/II-1ZC/III-1AC). The DNA origami scaffold (I-1CG/II-1ZC/III-1AC), in which each cavity contained a single site for one MA. The stem loops in red, green, and blue denote the binding sites for CLIP-G (CG) in cavity I, ZF-CLIP (ZC) in cavity II, and AZ-CLIP (AC) in cavity III, respectively. (b–e) AFM images of the DNA scaffold reacted with MAs. (b–d) The DNA scaffold (5 nM) was incubated with MAs (250 nM) (b) CLIP-G, (c) ZF-CLIP, or (d) AZ-CLIP for 30 min at ambient temperature. (e) One-pot coassembly reaction of three MAs on the DNA scaffold (I-1CG/II-1ZC/III-1AC). The DNA scaffold (5 nM) was incubated with CLIP-G, ZF-CLIP and AZ-CLIP (250 nM each) for 30 min at ambient temperature. These reactions were carried out in a buffer (pH 8.0) containing 40 mM Tris–HCl, 20 mM acetic acid, 12.5 mM MgCl_2_, 200 mM NaCl, 1 mM DTT, 1 μM ZnCl_2_, and 0.02% Tween 20. The reaction mixture was purified by size-exclusion chromatography and then analysed by atomic force microscopy (AFM). Yields at each cavity and coassembly yields were estimated by counting the number of cavities occupied by the modular adaptors (Table S14†).

Three modular adaptors, ZF-CLIP, AZ-CLIP, and CLIP-G, were reacted at the designed positions on the DNA scaffold. Rectangular DNA scaffold (Fig. 5a and S12†) containing three cavities was prepared as described previously.^18^ Each modular adaptor (250 nM) was incubated with 5 nM of the DNA scaffold (I-1CG/II-1ZC/III-1AC) containing a single binding site for CLIP-G, ZF-CLIP, and AZ-CLIP at cavity I, II, and III, respectively, in a buffer containing 200 mM NaCl (Fig. 5 and S13†). The reaction at the matched positions achieved over 90% yield in 30 min with less than 7% modifications at unmatched positions (Fig. 5b–d). Sequential reactions in the presence of 200 mM NaCl in the order of CLIP-G, AZ-CLIP, and ZF-CLIP confirmed that modification had occurred at the unmatched positions (less than 10% in total), as shown in Fig. S14.† The one pot co-assembly reaction of the modular adaptors for their respective target positions on the DNA scaffold (Fig. 5e) resulted in 87% coassembly yield after 30 min of incubation.

## Discussion

Based on eqn (1) of *k*_app_ in Fig. 1b, the factors affecting the kinetics and selectivity of the cross-linking reaction are the reactivity of the self-ligating protein tag (*k*_cov_) and the recognition process of the DNA binding domain (*k*_on_, *k*_off_, or *K*_D_). In our previous study, orthogonal cross-linking reactions of modular adaptors were mainly conducted by taking advantage of the chemoselectivity of protein tags (Fig. 1c).^15^ Thus, the selective reactions were driven by differences in the *k*_cov_ of the reaction. Although this strategy was effective for the orthogonal cross-linking reactions, only a few variations in the orthogonal modular adaptors were available because the number of effective pairs of protein tags and substrates was limited. In contrast, DNA-binding proteins show a wide range of sequence selectivity and affinity for the recognition modules of modular adaptors.^28^ In case 2 of eqn (1), where *k*_cov_ ≪ *k*_off_, the difference in *K*_D_ values of the matched and unmatched DNA binding complexes are a critical factor for regulating the selectivity of modular adaptors with the same protein tag to a given substrate in a defined DNA sequence. At the same *k*_cov_ of the protein tag module, the lower *K*_D_ of the sequence specific DNA-binding complex provides a higher rate constant for the matched pair (*k*_app_) and the higher *K*_D_ for the unmatched complex results in a lower rate constant for the unmatched pair 
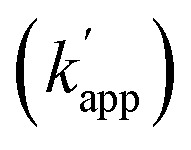
 for the sequence-selective modular adaptors. Based on the simulation of the kinetic scheme in Fig. 1b (Fig. S1†), the cross-linking reaction is expected to be orthogonal when the ratio of rate constants 
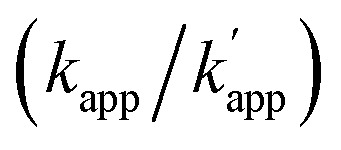
 is larger than 100. The zinc finger proteins used in this study, zif268 and AZP4, showed a nearly two orders of magnitude difference in *K*_D_ for complexes with matched and unmatched DNA sequences (Table S2†). In fact, modular adaptors with CLIP-tag and BC, developed in this study as the optimized protein tag and substrate pair for achieving *k*_cov_ ≪ *k*_off_, showed an orthogonality of 
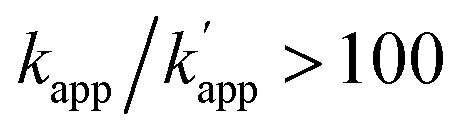
 while retaining their reactivities. The GCN4 derived homodimeric modular adaptor, CLIP-G, also revealed orthogonality to ZF-CLIP and AZ-CLIP.

Sequence specificity of the DNA binding protein has been shown to improve in the presence of a high concentration of NaCl, as nonspecific electrostatic interactions between the protein and DNA decreases with increasing salt concentration.^26^,^27^ Although the affinity of proteins for the target DNA sequence is also reduced in high concentrations of NaCl, selectivity between the target and non-target DNA is enhanced. Indeed, the *K*_D_ in the presence of 200 mM NaCl was significantly higher for both matched and unmatched complexes (Fig. S6 and Table S4†), and *k*_app_ showed nearly two-fold reduction (Fig. S7 and Table S6†), *i.e.*, for the matched combination of ZF-CLIP and ODN-ZF-BC: *k*_app_ (no NaCl) = 7.1 × 10^5^ M^–1^ s^–1^ and *k*_app_ (200 mM NaCl) = 3.1 × 10^3^ M^–1^ s^–1^; for the unmatched combination of ZF-CLIP and ODN-AZ-BC: 
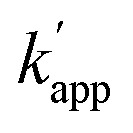
 (no NaCl) = 5.3 × 10^3^ M^–1^ s^–1^ and 
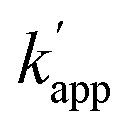
 (200 mM NaCl) < 10 M^–1^ s^–1^). Notably, the reactivity of the protein tag with its substrate was not influenced by the presence of NaCl (Fig. S10 and Tables S10, S11†). The sequence specificity of the crosslinking reaction was further studied in detail by using single or two nucleotide mutated ODN-ZF-BC for ZF-CLIP in the presence of 200 mM NaCl (Table S7†). A single nucleotide mutation in the consensus sequence of ODN-ZF-BC caused more than one order of magnitude difference in the apparent reaction rate constants (Fig. S9 and Table S9†), which was consistent with the difference between the *K*_D_ value for the complex of ZF-CLIP with ODN-ZF and that with the mutated sequences (Fig. S8 and Table S8†). These results strongly support our strategy in which the sequence selectivity of the cross-linking reaction by the modular adaptor was governed by *K*_D_ under the condition of case 2 of eqn (1) (Fig. 1b). Interestingly, the cross-linking reaction rate of the modular adaptor was drastically accelerated on the DNA origami scaffold compared to the reaction using oligo DNA. In the presence of 200 mM NaCl, AZ-CLIP and ODN-AZ-BC reacted, showing a *k*_app_ of 3.1 × 10^2^ M^–1^ s^–1^, which was the slowest combination among the matched pairs of the CLIP-type modular adaptors (Table S6†). AZ-CLIP rapidly reacted with BC in the matched sequence on the DNA origami scaffold while retaining selectivity and showed a *k*_app_ of 1.2 × 10^4^ M^–1^ s^–1^, which was nearly two orders of magnitude higher than that for the reaction with ODN-AZ-BC (Fig. S13†). This increase in the stability of the complex between the DNA-binding protein and DNA may arise from the large and negatively charged surface of the DNA origami scaffold surrounding the protein–DNA complex.^29^

## Conclusion

In summary, we have proved a design principle for sequence-specific DNA modifiers driven by specific DNA recognition based on the kinetic parameters for the DNA binding and modification reaction. The kinetic requirements for the complex formation step of DNA and the DNA binder as well as the successive proximity-driven intermolecular chemical reaction were applied to design sequence-selective modular adaptors by using appropriate combinations of a self-ligating protein tag and sequence-specific DNA-binding protein. The modular adaptors with the same self-ligating protein tag bearing different types of sequence-specific DNA-binding proteins indeed reacted with the substrate located in the respective matched DNA sequence, showing low reactivity with substrates in the unmatched sequence. Our design principle is useful for expanding the variation of sequence-specific modular adaptors by simply conjugating various types of sequence-specific DNA binding proteins^28^ to the same protein tag that reacts with an appropriate substrate. The resulting orthogonal set of modular adaptors can be used to add a variety of enzymes or receptors to a DNA scaffold to study the chemistry of spatially arranged biomolecules. More importantly, our design principle based on kinetic characteristics of selective modifiers can be applied for designing a wide variety of recognition-driven modifiers targeting not only DNA but also proteins and other biologically relevant molecules.

## Experimental procedures

### Materials

Single-stranded M13mp18, restriction enzymes (*Nde*I and *Hind*III), and BC-GLA-NHS (S9237S) were purchased from New England Biolabs (Ipswich, MA, USA). The *p*CLIP_f_(T7)-2 vector was generously donated by New England Biolabs. Purified oligonucleotides as the staple strands for DNA origami, oligonucleotide primers for gene construction, and all other oligonucleotides were obtained from Sigma-Aldrich (St. Louis, MO, USA), Thermo Fisher Scientific (Waltham, MA, USA), or Gene Design (Osaka, Japan). *Escherichia coli* BL21 (DE3) competent cells were purchased from Invitrogen (Carlsbad, CA, USA). A Mini Elute gel extraction kit was obtained from QIAGEN (Hilden, Germany). The HiTrap SP XL cation exchange column (5 mL), HisTrap HP column (5 mL), and Sephacryl S-400 were from GE Healthcare (Little Chalfont, UK). PrimeSTAR HS DNA polymerase, T4 DNA ligase, and *E. coli* DH5α competent cells were obtained from TaKaRa Bio (Shiga, Japan). Ultrafree-MC-DV was obtained from Merck Millipore (Billerica, MA, USA). A Cosmosil 5C18-MS II column (4.6 i.d. × 150 mm) and HPLC-grade acetonitrile were purchased from Nacalai Tesque (Kyoto, Japan). Gel electrophoresis-grade acrylamide, bis(acrylamide), phenol, and all other chemicals and reagents were purchased from Wako Chemicals (Osaka, Japan) or Nacalai Tesque. Phosphate buffer (PB) was prepared by mixing 20 mM Na_2_HPO_4_ and 20 mM NaH_2_PO_4_.

### Preparation of protein-tag derivatives (ZF-SNAP, AZ-SNAP, ZF-CLIP, AZ-CLIP, and CLIP-G)

Overexpression and purification of ZF-SNAP, AZ-SNAP, and AZ-CLIP were described in a previous report.^14^,^15^ ZF-CLIP and CLIP-G were newly designed in this study. As a typical example, the preparation of ZF-CLIP is shown below. All vectors encoding protein tag derivatives (pET-30a-ZF-CLIP, pET-30a-CLIP-G) were prepared and the protein tag derivatives (ZF-CLIP and CLIP-G) were overexpressed and purified in the same manner.

### Construction of vectors for ZF-CLIP (pET-30a-ZF-CLIP)

CLIP-tag in the *p*CLIP-tag (T7)-2 vector was amplified by PCR using the following primer pairs.

Forward primer (F_EcoRI_CLIP):

5′-TAATAAGAATTCGGCGGCTCCGGCGGCTCCGACAAAGATTGCGAAA-3′

Reverse primer (R_CLIP_HindIII):

5′-TTATTAAAGCTTTTAATGATGGTGATGATGATGGTGATGATGGTGGGTACCATTAACCTCGAGCCCGGGG-3′

The PCR products were separated on 1% agarose gel (TAE) and purified with a Mini Elute gel extraction kit. The PCR products and pET-30a-cys-zif268 were digested with *Eco*RI and *Hind*III and were purified in the same manner. These products were incubated with T4-DNA-ligase. The mixture was transferred into *E. coli* DH5α competent cells for amplification. The vector encoding ZF-CLIP (named pET-30a-ZF-CLIP) was purified, sequenced, and transferred into *E. coli* BL21(DE3) competent cells.

### Overexpression and purification of ZF-CLIP

The transformed cells were grown at 37 °C until the OD_600_ reached 0.5, and protein expression was induced with 1 mM IPTG for 24 h at 25 °C. The soluble fraction of the cell lysate containing the recombinant protein was loaded onto a HisTrap HP column equilibrated with 50 mM phosphate buffer (pH 8.0) containing 200 mM NaCl and 1 mM DTT and then eluted over an imidazole gradient. The main fractions containing the target protein were collected, loaded onto a HiTrap SP HP column equilibrated with 50 mM phosphate buffer (pH 7.0) containing 1 mM DTT, and then eluted over an NaCl gradient. The purified protein was dialyzed by using 50 mM phosphate buffer (pH 8.0) containing 1 mM DTT, 50 μM ZnCl_2_, and 50% glycerol and stored at –20 °C. The purity of the target protein was checked by SDS-PAGE. The major band on the gel corresponded to the calculated molecular weight with an estimated purity of over 95% (Fig. S15†). The modular adaptors were characterized by MALDI-TOF mass spectrometry (AXIMA-LNR, SA matrix, Shimadzu, Kyoto, Japan). ZF-CLIP: *m*/*z* calcd 32 462, observed 32 440; CLIP-G: *m*/*z* calcd 27 108, observed 27 083. Amino acid sequences and calculated molecular weights of the recombinant proteins used in this study are shown in Table S15.†

### Preparation of substrate-modified ODNs and Alexa 488 modified ODNs

A coupling reaction between amino-modified ODNs (100 μM) and Alexa Fluor 488 NHS ester or succinimidyl derivative of protein tag substrates (1 mM) was carried out in 100 mM phosphate buffer (pH 8.0) for 8 h at ambient temperature. The modified ODNs were purified by reversed-phase HPLC on a Cosmosil 5C18-MS II column (4.6 × 150 mm, eluted with 100 mM triethylammonium acetate buffer, pH 7.0, with a linear gradient over 30 min from 2.5% to 30% acetonitrile at flow rate of 1.0 mL min^–1^) and characterized by MALDI-TOF mass spectrometry (AXIMA-LNR, Shimadzu, HPA matrix). A488-ODN-ZF: *m*/*z* calcd 11 397, observed 11 395; A488-ODN-AZ: *m*/*z* calcd 11 400, observed 11 399; A488-ODN-AP: *m*/*z* calcd 12 011, observed 12 014. ODN-AP-2BC: *m*/*z* calcd 12 290, observed 12 290, ODN-8G-AP-2BC: *m*/*z* calcd 21 577, observed 21 578, ODN-11G-ZF-BC: *m*/*z* calcd 21 160, observed 21 167, ODN-ZF-BC: *m*/*z* calcd 14 287, observed 14 287, ODN-ZF(G/T)-BC: *m*/*z* calcd 14 286, observed 14 289, ODN-ZF(G/C)-BC: *m*/*z* calcd 14 287, observed 14 282, ODN-ZF(GG/TC)-BC: *m*/*z* calcd 14 286, observed 14 288, and ODN-ZF(GG/TC)-BC: *m*/*z* calcd 14 286, observed 14 291. The method for preparing ODN-AZ-BC, ODN-AZ-BG, and ODN-24D-AZ-BC was described in a previous report.^15^

### Preparation of ^32^P-end-labeled ODNs and analyses of covalent-linkage formation between ^32^P-end-labeled ODNs and modular adaptors

The ODNs were 5′-^32^P-end-labeled as previously described.13*b* In a typical experiment for kinetic analysis, ^32^P-ODN-ZF-BC (less than 0.5 nM) was incubated with ZF-CLIP (10 nM) in a buffer (pH 8.0) containing 40 mM Tris–HCl, 20 mM acetic acid, 12.5 mM MgCl_2_, 1 mM DTT, 1 μM ZnCl_2_, 0.02% Tween 20, 200 nM BSA, and 100 nM calf thymus DNA, with or without 200 mM NaCl at ambient temperature. Aliquots were collected at defined reaction times and quenched by adding SDS and formamide. The aliquots were analysed by 8 M urea PAGE and the intensities of mobility-shifted bands on the gel were analysed with a Storm 860 molecular imager (Amersham plc, Amersham, UK). The kinetics data were fitted to a reaction model assuming first-order kinetics (eqn (1)), and then the second-order rate constants 
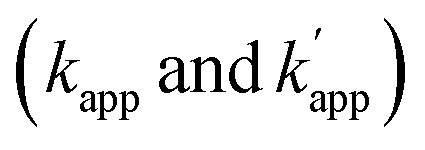
 were obtained by dividing the determined first-order rate constants by the concentration of modular adaptors.

1 *Y* = 1 – *e*^–*kt*^

*Y*, *k* and *t* represent the fraction of the cross-linked product, pseudo-first-order rate constant and reaction time, respectively.

To evaluate orthogonality, each 5′-^32^P-end-labeled ODN (ODN-ZF-BC, ODN-AZ-BC and ODN-AP-2BC) was incubated with a modular adaptor (10 or 100 nM) in a buffer (pH 8.0) containing 40 mM Tris–HCl, 20 mM acetic acid, 12.5 mM MgCl_2_, 1 mM DTT, 1 μM ZnCl_2_, 0.02% Tween 20, 200 nM BSA, and 100 nM calf thymus DNA, with or without 200 mM NaCl at ambient temperature.

### Preparation of the DNA origami scaffold with three cavities

A solution (50 μL) containing M13mp18 single-stranded DNA (New England Biolabs, 10 nM) and staple DNA strands (5 equiv., 50 nM, nucleotide sequences of all staple DNA strands are described in our previous report^18^ and Table S13†) in buffer (40 mM Tris–HCl, 20 mM acetic acid, 12.5 mM MgCl_2_, pH 8.0) was heated at 95 °C for 1 min, annealed at 53 °C for 30 min, and held at 4 °C in a thermal cycler. The samples were purified by size-exclusion chromatography (400 μL volume of Sephacryl S-400, GE Healthcare) equilibrated with a buffer (pH 8.0) containing 40 mM Tris–HCl, 20 mM acetic acid, and 12.5 mM MgCl_2_ in Ultrafree-MC-DV (Millipore).

### Preparation of the DNA origami scaffold assembled with modular adaptors

A DNA origami scaffold was incubated with ZF-CLIP, AZ-CLIP, and/or CLIP-G under the conditions given in the captions of the figures or tables. For example, the cross-linking reaction of each of modular adaptor was carried out for 30 min at ambient temperature with 5 nM DNA scaffold and 250 nM modular adaptor (ZF-CLIP, AZ-CLIP, or CLIP-G) in buffer (pH 8.0) containing 40 mM Tris–HCl, 20 mM acetic acid, 12.5 mM MgCl_2_, 1 mM DTT, 1 μM ZnCl_2_, 200 mM NaCl, and 0.02% Tween 20. The mixture was purified by size-exclusion chromatography (400 μL volume of Sephacryl S-400 in Ultrafree-MC-DV) equilibrated with the buffer used for the reaction to remove unbound modular adaptors. The fractions containing DNA origami scaffolds were utilized for AFM analyses.

### AFM imaging and statistical analysis

The sample was deposited on a freshly cleaved mica (1.5 mm diameter) surface and adsorbed for 5 min at ambient temperature, followed by three washes with a buffer (pH 8.0) containing 40 mM Tris–HCl, 20 mM acetic acid, and 12.5 mM MgCl_2_. The sample was scanned in tapping mode using a fast-scanning AFM system (Nano Live Vision, RIBM Co., Ltd., Tsukuba, Japan) with a silicon nitride cantilever (Olympus BL-AC10DS-A2, Tokyo, Japan). At least three independent preparations of each sample were analyzed by AFM and several images were acquired from different regions of the mica surface. The total number of DNA scaffolds corresponds to the number of expected rectangular shapes possessing three cavities observed by AFM. Specific and nonspecific binding of modular adaptors was counted for only the modular adaptor bound to the perfectly folded DNA scaffold. The yield of the DNA-scaffold-assembled modular adaptor was calculated as described previously^14^,^18^ and the results are shown in Table S14.†

### Fluorescence polarization spectroscopy

A fluorescence polarization assay by using an Infinite 200 PRO F Plex (Tecan, Männedorf, Switzerland) was conducted to determine the equilibrium dissociation constant (*K*_D_) (Fig. S4, S6 and S8†) and kinetic parameter (*k*_on_ and *k*_off_) (Fig. S11†) for the complexes of the modular adaptor with a fluorophore-modified ODN. The rate constant of covalent bond formation of the protein tag derivatives and fluorophore-modified substrates was also determined (Fig. S10†). The kinetic parameter *k*_obs_ (s^–1^) was fitted to the reaction model assuming first-order kinetics, and then the second-order rate constants of the process were determined. These assays were carried out in a buffer (pH 8.0) containing 40 mM Tris–HCl, 20 mM acetic acid, 12.5 mM MgCl_2_, 1 mM DTT, 1 μM ZnCl_2_, 100 nM calf thymus DNA, and 0.02% Tween 20 at 25 °C with or without 200 mM NaCl.

## Conflicts of interest

The authors have no conflicts of interest directly relevant to the content of this article.

## Supplementary Material

Supplementary information
