# Supplementary material for: Rational design of a DNA sequence-specific modular protein tag by tuning the alkylation kinetics
Source: Chem Sci. 2019 Aug 20;10(40):9315–25. doi: 10.1039/c9sc02990g (PMC7006624; doi:10.1039/c9sc02990g)
Supplement: Supplementary file 1 [file SC-010-C9SC02990G-s001.pdf]

Electronic Supplementary Material (ESI) for Chemical Science.
This journal is © The Royal Society of Chemistry 2019

SUPPORTING INFORMATION

Rational Design of a DNA Sequence-Specific Modular Protein Tag by Tuning the Alkylation Kinetics

Thang Minh Nguyen, Eiji Nakata, Zhengxiao Zhang, Masayuki Saimura, Huyen Dinh, and Takashi Morii*

Institute of Advanced Energy, Kyoto University, Uji, Kyoto 611-0011, Japan.

*To whom correspondence should be addressed:

Prof. Takashi Morii

Tel.: +81 774-38-3585

Fax: +81 774-38-3516

E-mail: t-morii@iae.kyoto-u.ac.jp

Contents

Table S1-S15	s3-s7
Figure S1-S15	s8-s22
Reference	s23

Table S1 Nucleotide sequences of Alexa Fluor 488 (A488) modified ODN used to determine equilibrium disassociation constants (K_D), association (k_{on}) and dissociation (k_{off}) rate constants for the complexes of modular adaptors with ODN by fluorescence polarization measurements.

**Oligo name**	**Sequence (from 5' to 3')** T^A = A488 modified T
A488-ODN-ZF	CGCGACGCCCACGCGCGTT^{A}TTCGCGCGTGGGCGTCGC
A488-ODN-AZ	CGCGATGCCACGTAGCGTT^{A}TTCGCTACGTGGCATCGC
A488-ODN-AP	CGTTCATGACTCATGAGTT^{A}TTCTCATGAGTCATGAAC

Table S2 Equilibrium disassociation constants (K_D) for the complexes of modular adaptors with ODN determined by fluorescence polarization measurements.

Recognition module	K_D (nM)		
	ODN-ZF	ODN-AZ	ODN-AP
ZF-CLIP	**8.1 ± 0.6**	> 1000	> 1000
AZ-CLIP	> 1000	**74 ± 3.0**	n.d.
CLIP-G	> 1000	> 1000	**5.1 ± 0.5**

n.d. not determined

Table S3 Kinetic parameters for the cross-linking reaction between 5'-^{32}P-end-labeled BC-modified ODN derivatives and modular adaptors containing CLIP-tag. Rate constants for the matched pairs (k_{app}) are in bold, and for the unmatched pairs (k'_{app})) in plane.

**Rate constant (M^{-1} s^{-1})**	**k_{app} (M^{-1} s^{-1}) (ODN-S)**	k'_{app} (M^{-1} s^{-1}) (ODN-S)	
**ZF-CLIP**	**(7.1 ± 0.2) × 10^5 (ODN-ZF-BC)**	(2.8 ± 0.2) × 10^3 (ODN-AZ-BC)	(1.5 ± 0.3) × 10^2 (ODN-AP-2BC)
**AZ-CLIP**	**(5.0 ± 0.7) × 10^5* (ODN-AZ-BC)**	(8.1 ± 2.7) × 10^2 (ODN-ZF-BC)	(5.8 ± 1.5) × 10^2 (ODN-AP-2BC)
**CLIP-G**	**(2.1 ± 0.1) × 10^6 (ODN-AP-2BC)**	(1.7 ± 0.4) × 10^3 (ODN-ZF-BC)	(5.6 ± 0.4) × 10^3 (ODN-AZ-BC)

* taken from ref. 15.

Table S4 Equilibrium disassociation constants (K_D) in the presence of 200 mM NaCl for the complexes of modular adaptors with oligo DNA determined by fluorescence polarization.

Recognition module	K_D (nM)		
	ODN-ZF	ODN-AZ	ODN-AP
ZF-CLIP	**> 1000**	n.d.	n.d.
AZ-CLIP	n.d.	**> 1000**	n.d.
CLIP-G	n.d.	n.d.	**> 1000**

n.d. not determined

Table S5 Kinetic parameters for the cross-linking reaction between 5'-^{32}P-end-labeled BG-modified ODN with modular adaptors containing SNAP-tag in the presence of 200 mM NaCl. Rate constants for the matched pair (k_{app}) are in bold, and for the unmatched pairs (k'_{app}) in plane

**Rate constant (M^{-1} s^{-1})**	**k_{app} (M^{-1} s^{-1}) (ODN-S)**	k'_{app} (M^{-1} s^{-1}) (ODN-S)
**ZF-SNAP**	**(1.3 ± 0.2) × 10^4 (ODN-ZF-BG)**	(6.1 ± 0.3) × 10^2 (ODN-AZ-BG)
**AZ-SNAP**	**(2.2 ± 0.2) × 10^4 (ODN-AZ-BG)**	(8.5 ± 0.3) × 10^2 (ODN-ZF-BG)

Table S6 Kinetic parameters for the cross-linking reaction between 5'-^{32}P-end-labeled BC-modified ODN derivatives with modular adaptors containing CLIP-tag in the presence of 200 mM NaCl. Rate constants for the matched pairs are in bold, and the unmatched pairs in plane.

**Rate constant (M^{-1} s^{-1})**	**k_{app} (M^{-1} s^{-1}) (ODN-S)**	k'_{app} (M^{-1} s^{-1}) (ODN-S)	
**ZF-CLIP**	**(3.1 ± 0.2) × 10^3 (ODN-ZF-BC)**	< 10 (ODN-AZ-BC)	< 10 (ODN-AP-2BC)
**AZ-CLIP**	**(3.1 ±0.2) × 10^2 (ODN-AZ-BC)**	< 10 (ODN-ZF-BC)	< 10 (ODN-AP-2BC)
**CLIP-G**	**(1.8 ± 0.3) × 10^4 (ODN-AP-2BC)**	< 10 (ODN-ZF-BC)	< 10 (ODN-AZ-BC)

Table S7 Nucleotide sequences of BC modified ODN-ZF derivatives.

**Oligo name**	**Sequence (from 5' to 3')** T^{BC} = BC modified T
ODN-ZF-BC	CGCGTATAACGCCCACGCGCGTT^{BC}TTCGCGCGTGGGCGTTATACGC
ODN-ZF(G/T)-BC	CGCGTATAACGCCAACGCGCGTT^{BC}TTCGCGCGTTGGCGTTATACGC
ODN-ZF(G/C)-BC	CGCGTATAAGGCCCACGCGCGTT^{BC}TTCGCGCGTGGGCCTTATACGC
ODN-ZF(GG/TC)-BC	CGCGTATAAGGCCAACGCGCGTT^{BC}TTCGCGCGTTGGCCTTATACGC
ODN-ZF(GC/CT)-BC	CGCGTATAACAGCCACGCGCGTT^{BC}TTCGCGCGTGGCTGTTATACGC

Table S8 Equilibrium disassociation constants (K_D) in the presence of 200 mM NaCl for the complexes of ZF-CLIP with A488-ODN-ZF derivatives determined by fluorescence polarization.

ODN-ZF derivatives	K_D (nM) ZF-CLIP	K_D / $K_{D(ODN-ZF)}$
ODN-ZF	56 ± 7.0 [a]	1
ODN-ZF(G/T)	(1.1 ± 0.2) × 10^3 [b]	19.6
ODN-ZF(G/C)	(6.5 ± 0.7) × 10^2 [b]	11.6
ODN-ZF(GG/TC)	> 10^4 [b]	> 200
ODN-ZF(GC/CT)	> 10^4 [b]	> 200

[a] obtained from the direct titration in florescence polarization measurement.

[b] obtained from the competitive titration in florescence polarization measurement.

Table S9 Kinetic parameters for the cross-linking reaction between 5'-^{32}P-end-labeled BC-modified ODN-ZF-BC derivatives with and modular adaptors containing CLIP-tag in the presence of 200 mM NaCl.

ODN-ZF-BC derivatives	k_{app} or k'_{app} (M^{-1} s^{-1})	k_{app} / k'_{app}
	ZF-CLIP	
ODN-ZF-BC	(5.1 ± 0.6) × 10^4	-
ODN-ZF(G/T)-BC	(1.6 ± 0.2) × 10^3	32
ODN-ZF(G/C)-BC	(2.3 ± 0.2) × 10^3	22
ODN-ZF(GG/TC)-BC	n.d.	> 1000
ODN-ZF(GC/CT)-BC	n.d.	> 1000

Table S10 Kinetic parameters (k_{cross}) for the cross-linking reaction between CLIP-tag and SNAP-tag derivatives with ATTO488-BC and ATTO488-BG, respectively.

k_{cross} ($M^{-1}s^{-1}$)	ATTO488-BG	ATTO488-BC
**ZF-SNAP**	(6.0 ± 0.1) × 10^3	< 10
**AZ-SNAP**	(2.4 ± 0.1) × 10^4	< 10
**ZF-CLIP**	< 10	87 ± 7.0
**AZ-CLIP**	< 10	70 ± 1.7
**CLIP-G**	< 10	83 ± 1.5

Table S11 Kinetic parameters (k_{cross}) for the cross-linking reaction between CLIP-tag and SNAP-tag derivatives with ATTO488-BC and ATTO488-BG, respectively, in the presence of 200 mM NaCl.

k_{cross} ($M^{-1}s^{-1}$)	ATTO488-BG	ATTO488-BC
**ZF-SNAP**	(5.7 ± 0.8) × 10^3	< 10
**AZ-SNAP**	(1.4 ± 0.2) × 10^4	< 10
**ZF-CLIP**	< 10	21 ± 6.7
**AZ-CLIP**	< 10	32 ± 0.4
**CLIP-G**	< 10	23 ± 0.6

Table S12 Association (k_{on}) and dissociation (k_{off}) rate constants for the complexes of modular adaptors with ODN determined by fluorescence polarization measurements.

Modular adaptor	ODN	k_{on} ($M^{-1}s^{-1}$)	k_{off} (s^{-1})	K_D (nM)[a]
ZF-CLIP	A488-ODN-ZF	1.3×10^6	4.3×10^{-2}	31 [*2]
	A488-ODN-AZ	n.d. (1.3×10^6 [*1])	n.d. (1.3 [*1])	1000 [*1]

[a] obtained from the rate constants: $k_{off} / k_{on} = K_D$

n.d. not determined

[*1] The k_{off} value for the complex of ZF-CLIP and ODN-AZ was not determined because of the fast kinetics under our experimental condition ($k_{off} < 1.0$ s^{-1}). Thus, k_{off} for the complex between ZF-CLIP and ODN-AZ was calculated from its K_D (1000 nM) deduced from the titration of fluorescence polarization (Table S2) and k_{on} (1.3×10^6 $M^{-1}s^{-1}$) because k_{on} of the unmatched complex is similar to that of the matched complex[S4,6-9].

[*2] K_D for the complex between ZF-CLIP and ODN-ZF was consistent with the value obtained from the titration of fluorescence polarization (Table S2).

Table S13 Nucleotide sequences of staple strands including zif268, AZP4 and GCN4 binding sites with BC modified T^{BC}. The GCN4, zif268 and AZP4 binding sites on the staple strands were colored in red, blue and green, respectively.

Oligo name	Sequence (from 5' to 3')
8g-AP-2BC	TAATACTGT^{BC}TCATGAGTCATGAGTTTTCT^{BC}CATGACTCATGAACCAGGCAAGGCAAAGACATCCAATA
11g-ZF-BC	AACAGGTCCTTACGCCCACGCGCGTT^{BC}TTCGCGCGTGGGCGTAAGGAACCAGACCGGAAGATTCGAGC
24D-AZ-BC	GGACAGATCTTATGCCACGTAGCGTT^{BC}TTCGCTACGTGGCATAAGAAATTGTGTCGAAATCTGTATCAT

Table S14 Total numbers and yields of the DNA scaffold assembled with the modular adaptors analysed by AFM.

DNA scaffold	Modular adaptor derivatives	Number of well-formed DNA scaffold	Numbers and yields of the modified cavities			Coassembly yield	AFM image
			Cavity I	Cavity II	Cavity III		
**I-1CG/II-1ZC/III-1AC** (5 min incubation with 200 mM NaCl)	AZ-CLIP	174	4 **[2%]**	6 **[4%]**	99 **[58%]**	-	Figure S13c
**I-1CG/II-1ZC/III-1AC** (10 min incubation with 200 mM NaCl)	AZ-CLIP	155	6 **[4%]**	10 **[6%]**	117 **[75%]**	-	Figure S13d
**I-1CG/II-1ZC/III-1AC** (20 min incubation with 200 mM NaCl)	AZ-CLIP	176	7 **[4%]**	7 **[4%]**	159 **[90%]**	-	Figure S13e
**I-1CG/II-1ZC/III-1AC** (sequential assembly with 200mM NaCl)	CLIP-G	142	131 **[92%]**	7 **[5%]**	8 **[6%]**	-	Figure S14c
	CLIP-G and AZ-CLIP	135	126 **[93%]**	12 **[9%]**	128 **[95%]**	124 **[92%]**	Figure S14d
	CLIP-G, AZ-CLIP and ZF-CLIP	144	134 **[93%]**	132 **[92%]**	135 **[94%]**	123 **[85%]**	Figure S14e
**I-1CG/II-1ZC/III-1AC** (30 min incubation with 200 mM NaCl)	CLIP-G	165	151 **[92%]**	10 **[6%]**	14 **[8%]**	-	Figure 5b
	ZF-CLIP	197	5 **[3%]**	177 **[90%]**	9 **[5%]**	-	Figure 5c
	AZ-CLIP	153	3 **[2%]**	7 **[5%]**	143 **[93%]**	-	Figure 5d, Figure S13f
**I-1CG/II-1ZC/III-1AC** (one-pot assembly with 200 mM NaCl)	CLIP-G, AZ-CLIP and ZF-CLIP	144	137 **[95%]**	131 **[91%]**	139 **[97%]**	125 **[87%]**	Figure 5e

Table S15 Amino acid sequences and molecular weights of modular adaptor derivatives.

**Derivatives**	**Amino acid sequences**	**Calculated molecular weight** (Da)
ZF-CLIP	MMKTGEKRPYACPVESCDRRFSRSDELTRHIRIHTGQKPFQCRICMRNFSRSDHL<br>TTHIRTHTGEKPFACDICGRKFARSDERKRHTKIHTGEKEFGGSGGSMDKDCEMK<br>RTTLDSPLGKLELSGCEQGLHRIIFLGKGTSAADAVEVPAPAAVLGGPEPLIQAT<br>AWLNAYFHQPEAIEEFPVPALHHPVFQQESFTRQVLWKLLKVVKFGEVISESHLA<br>ALVGNPAATAAVNTALDGNPVPILIPCHRVVQGDSDVGPYLGGLAVKEWLLAHEG<br>HRLGKPGLGGGSGGSHHHHHH	32462
CLIP-G	MDKDCEMKRTTLDSPLGKLELSGCEQGLHRIIFLGKGTSAADAVEVPAPAAVLGG<br>PEPLIQATAWLNAYFHQPEAIEEFPVPALHHPVFQQESFTRQVLWKLLKVVKFGE<br>VISESHLAALVGNPAATAAVNTALDGNPVPILIPCHRVVQGDSDVGPYLGGLAVK<br>EWLLAHEGHRLGKPGLGGGSGDPAALKRARNTEAARRSRARKLQRMKQLEDKVEE<br>LLSKNYHLENEVARLKKLVGERHHHHHH	27108

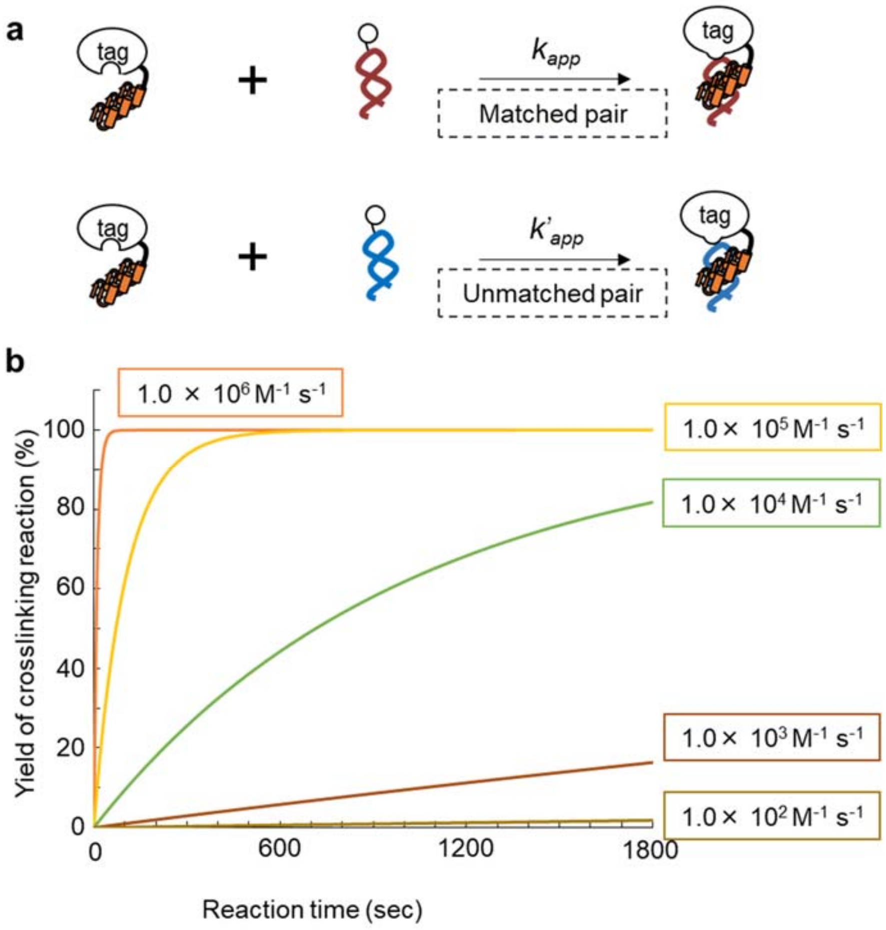

Figure S1 Simulation of the time-course changes in the yield of cross-linking reaction. (a) Schematic illustration for the cross-linking reactions of matched and unmatched pairs. The apparent reaction rate constants for the matched pair and the unmatched pair are indicated by k_{app} [$M^{-1}s^{-1}$] and k'_{app} [$M^{-1}s^{-1}$], respectively. (b) The simulation data for the time-course change in yield of cross-linking reaction. The yield of cross-linking reaction at the apparent reaction rates (10^2-10^6 $M^{-1}s^{-1}$) were simulated by using Tenua[S1–S3] as the kinetic simulator. The concentrations of substrate tethered ODN and modular adaptor were set to 10 nM and 100 nM, respectively.

By comparing the yields of cross-linking reaction for k_{app} and k'_{app}, the one order of magnitude difference of reaction rate is not sufficient to warrant the orthogonality for these cross-linking reactions. For example, when k_{app} was set to 1×10^5 $M^{-1}s^{-1}$, the yield of cross-linking reaction reached 100% within 10 min. When k'_{app} was 1×10^4 $M^{-1}s^{-1}$, the undesired cross-linking reaction proceeded to 50% at 10 min. When k'_{app} was 1×10^3 $M^{-1}s^{-1}$, the yield of undesired cross-linking reaction was less than 10% in the reaction time. These simulation results strongly indicated that the difference of more than two orders of magnitude between k_{app} and k'_{app} was required to warrant the orthogonality for these cross-linking reactions.

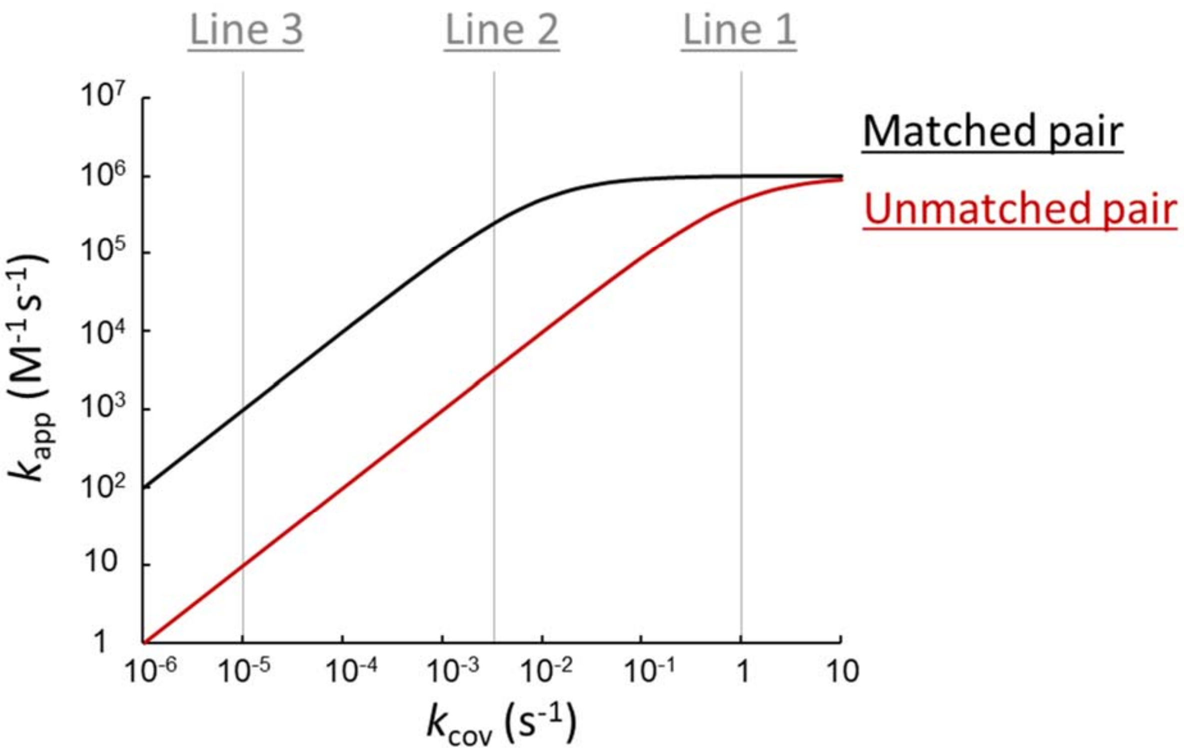

Figure S2 Simulation of the apparent reaction rates (k_{app}) ($M^{-1}s^{-1}$) with various rate constants for the covalent bond formation (k_{cov}) (10^{-6}-10 s^{-1}). As the simulation condition, the association rate constant (k_{on}) was set to 1 × 10^6 $M^{-1}s^{-1}$ for both matched and unmatched pair and the dissociation rate constant (k_{off}) was set to 0.01 s^{-1} for matched pair and 1 s^{-1} to unmatched pair, respectively. These parameters are comparable to the reported k_{on} *and* k_{off} values[S4] and our determined value from the fluorescence polarization analysis (Figure S11 and Table S12). The apparent reaction rates of matched (k_{app}) and unmatched (k'_{app}) pairs changed with varying the k_{cov} value. In the case of k_{cov} = 1 s^{-1} (Line 1), k_{app} is 1 × 10^6 $M^{-1}s^{-1}$ and k'_{app} is around 1 × 10^6 $M^{-1}s^{-1}$, thus the cross-linking reactions of matched and unmatched pairs proceed within the same time scale. In the case of k_{cov} = 1 × 10^{-5} s^{-1} (Line 3), k_{app} is 1 × 10^3 $M^{-1}s^{-1}$ and k'_{app} is around 10 $M^{-1}s^{-1}$, giving two orders of magnitude difference, but required long incubation time to complete the reaction (Figure S1). In the case of k_{cov} = 4 × 10^{-3} s^{-1} (Line 2), k_{app} is 3 × 10^5 $M^{-1}s^{-1}$ and k'_{app} is around 4 × 10^3 $M^{-1}s^{-1}$, showing almost two orders of magnitude difference with maintaining enough reactivity for the matched pair. These simulation results strongly indicate that the tuning the rate constant of covalent bond formation is an effective way to realize the sequence selectivity between matched and unmatched pairs.

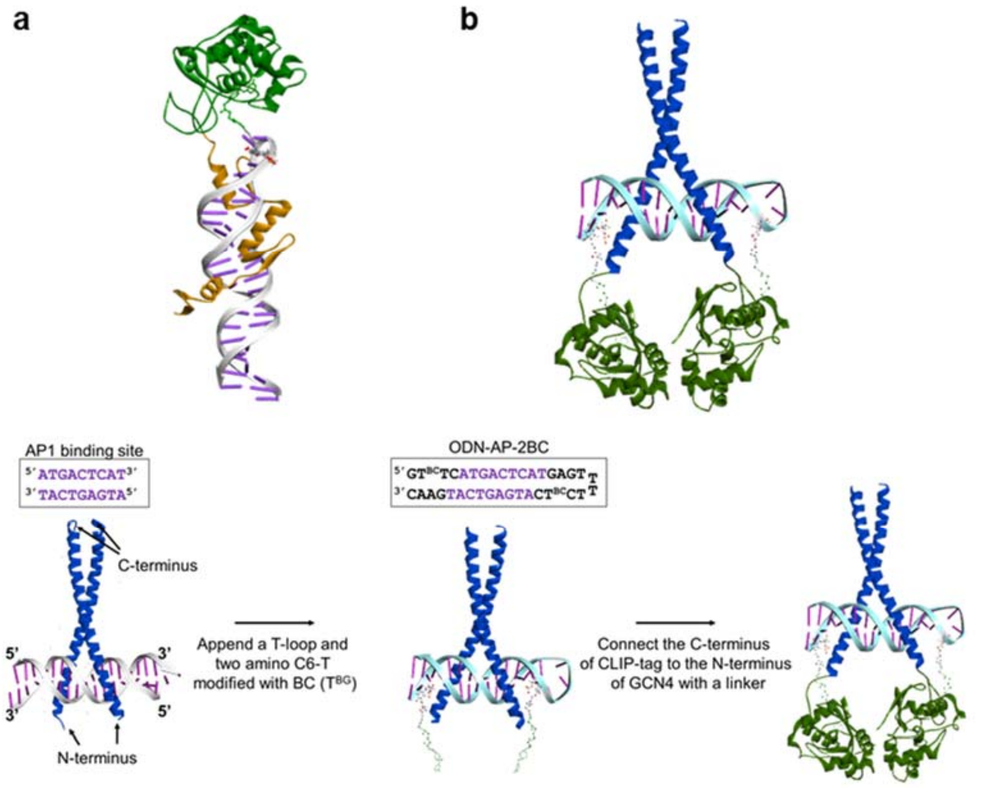

Figure S3 Molecular models for the complexes of (a) ZF-CLIP and ODN-ZF-BC and (b) CLIP-G and ODN-AP-2BC based on the crystal structures of the complexes between zif268 and ODN (PDB ID: 1ZAA), SNAP-tag (PDB ID: 3KZY), GCN4 and DNA containing AP1 sequence (PDB ID: 1DGC) and SNAP-tag (PDB ID : 3KZY). The model structure of CLIP-tag was obtained based on the structure of SNAP-tag.[S5] (b) A scheme illustrates the design of CLIP-G and ODN-AP-2BC. Two BCs were appended in the major groove of ODN-AP with 10 base-pairs inter-distance, which allows access of the introduced BCs to CLIP-tags of modular adaptor CLIP-G. The molecular models were constructed by using Discovery Studio (version 3.1, Accelrys Inc.).

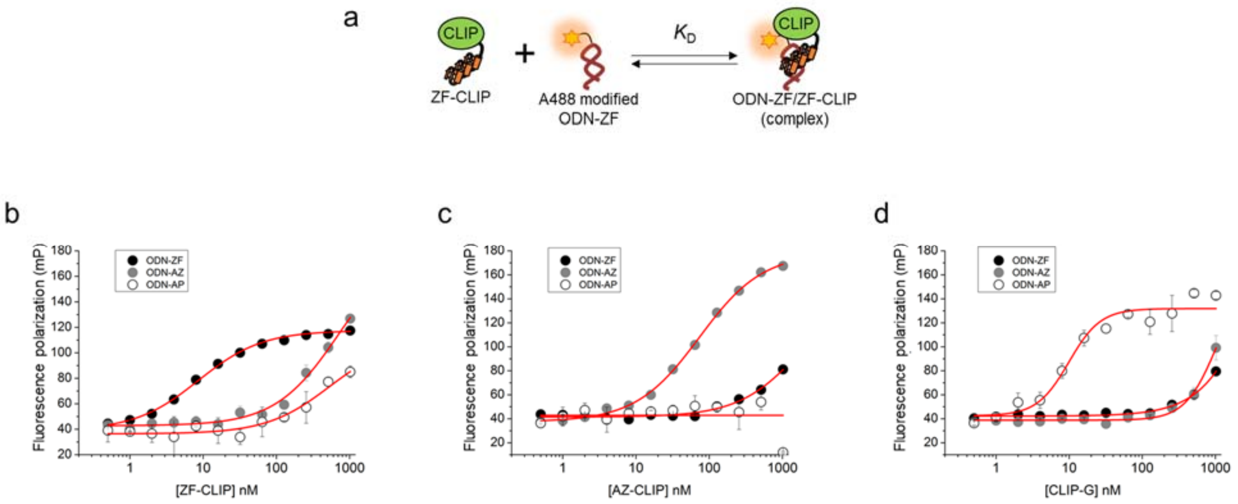

Figure S4 (a) A scheme illustrates fluorescence polarization analyses for the binding of modular adaptor to fluorophore modified ODN. Titration of (b) ZF-CLIP, (c) AZ-CLIP and (d) CLIP-G with the fluorophore modified ODNs. Reaction was conducted in a buffer (pH 8.0) containing 40 mM Tris-HCl, 20 mM acetic acid, 12.5 mM $MgCl_2$, 1 mM DTT, 1 μM $ZnCl_2$, 0.02% Tween20, 200 nM BSA, 100 nM calf thymus DNA and 0.1 nM fluorophore modified ODN (Table S1) with increasing concentrations of modular adaptor (0.5 nM to 1024 nM) at ambient temperature. Estimated equilibrium dissociation constants are listed in Table S2.

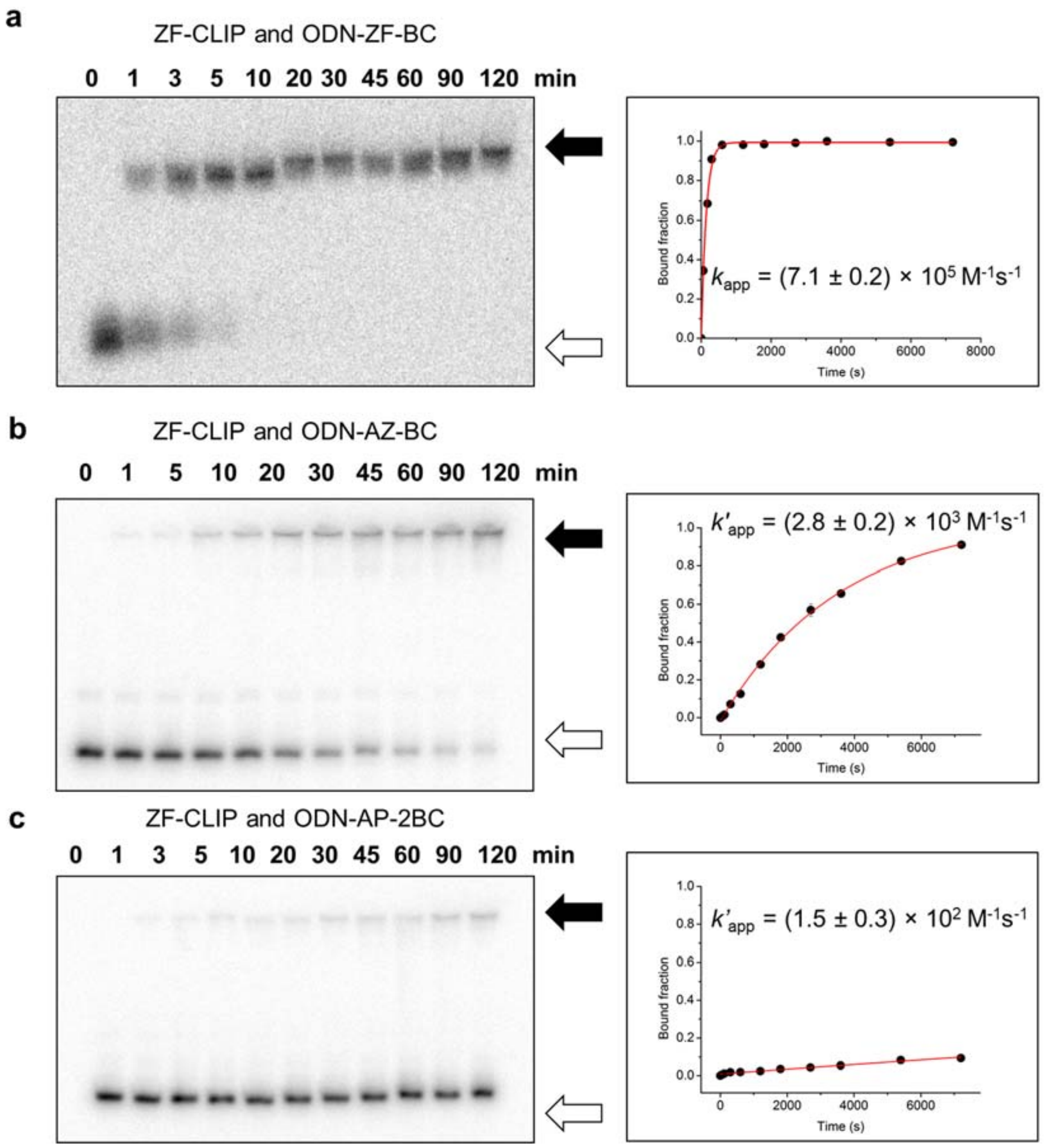

Figure S5 Autoradiogram show denaturing gel electrophoretic analyses of the cross-linking reactions of 5'-^{32}P-end-labeled ODN-ZF-BC, ODN-AZ-BC, and ODN-AP-2BC with (a) 10 nM ZF-CLIP and (b, c) 100 nM ZF-CLIP, respectively, (left). Open arrows and filled arrows denote free ODN and ZF-CLIP bound ODN, respectively. Time-course plots for the crosslinking reaction of ODN-ZF-BC, ODN-AZ-BC, and ODN-AP-2BC with ZF-CLIP to obtain the rate constants ($\boldsymbol{k}_{\mathbf{app}}$) (right). The determined rate constants are listed in Table S3. Reaction was conducted in a buffer (pH 8.0) containing 40 mM Tris-HCl, 20 mM acetic acid, 12.5 mM $MgCl_2$, 1 mM DTT, 1 μM $ZnCl_2$, 0.02% Tween20, 200 nM BSA, 100 nM calf thymus DNA and at ambient temperature.

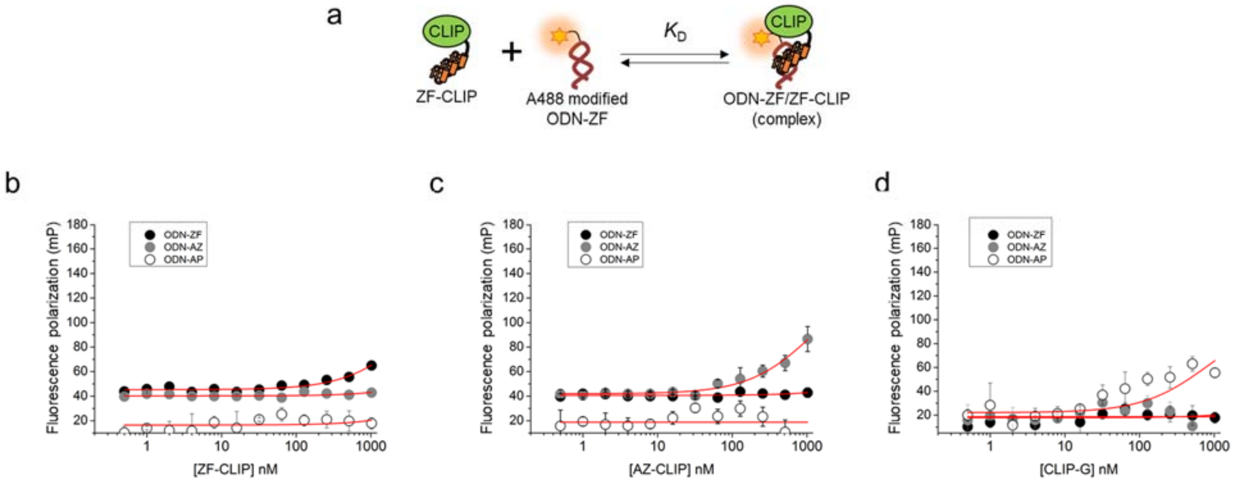

Figure S6 (a) A scheme illustrates fluorescence polarization analyses for the binding of modular adaptor to fluorophore modified ODN. Titration of (b) ZF-CLIP, (c) AZ-CLIP and (d) CLIP-G with the fluorophore modified ODNs <u>in the presence of 200 mM NaCl</u>. Reaction was conducted in a buffer (pH 8.0) containing 40 mM Tris-HCl, 20 mM acetic acid, 200 mM NaCl, 12.5 mM $MgCl_2$, 1 mM DTT, 1 μM $ZnCl_2$, 0.02% Tween20, 200 nM BSA, 100 nM calf thymus DNA and 0.1 nM fluorophore modified ODN with increasing concentrations of modular adaptor (0.5 nM to 1024 nM) at ambient temperature. Estimated equilibrium dissociation constants are listed in Table S4.

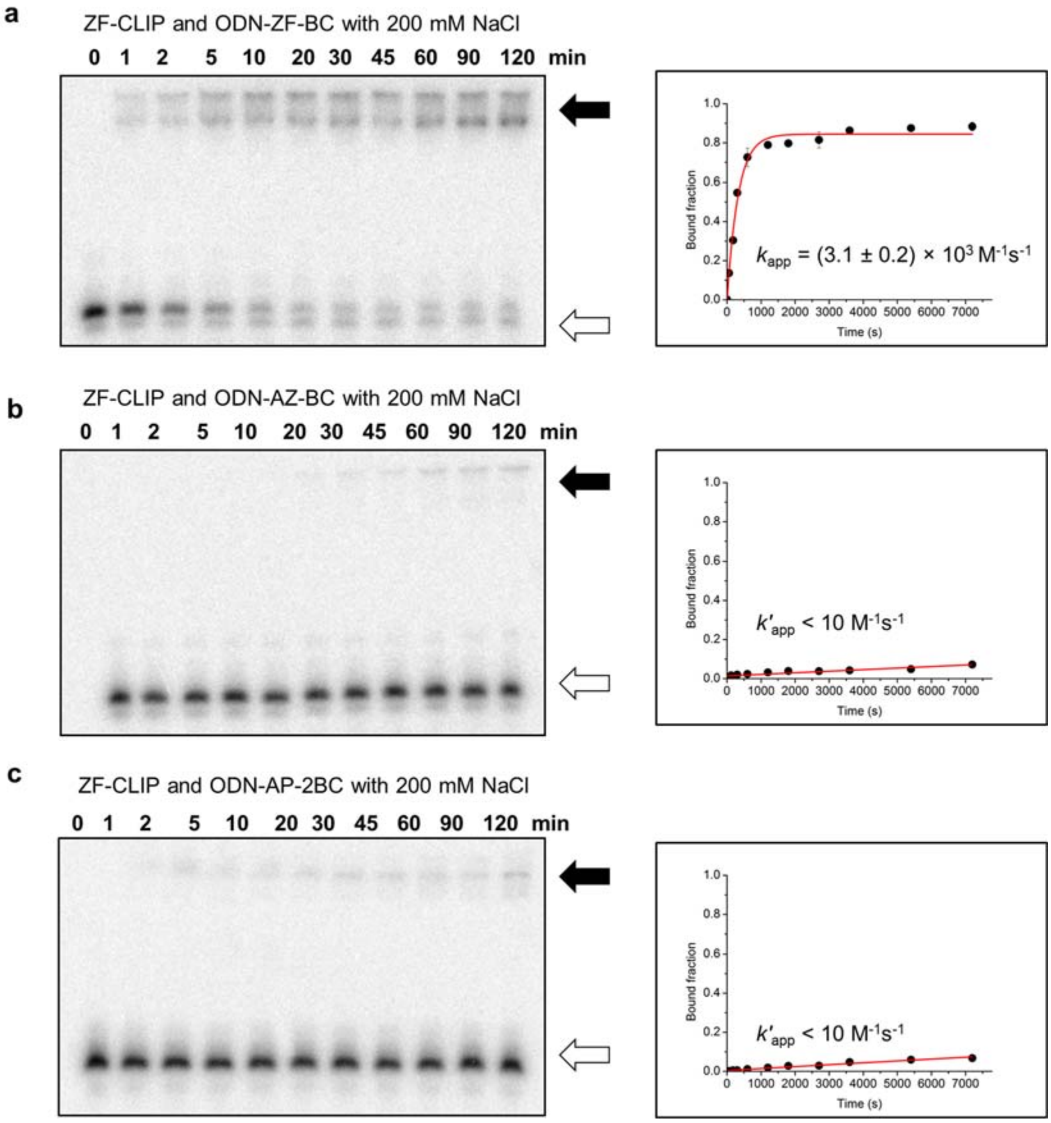

Figure S7 Autoradiogram show denaturing gel electrophoretic analyses of the cross-linking reactions of 5'-^{32}P-end-labeled ODN-ZF-BC, ODN-AZ-BC and ODN-AP-2BC with ZF-CLIP (100 nM) (left). Open arrows and filled arrows denote free ODN and ZF-CLIP bound ODN, respectively. Time-course plots for the crosslinking reaction of ODN-ZF-BC, ODN-AZ-BC, and ODN-AP-2BC with ZF-CLIP to obtain the rate constants ($\boldsymbol{k}_{\mathbf{app}}$) (right). The determined rate constants are listed in Table S5 and S6. Reaction was conducted in a buffer (pH 8.0) containing 40 mM Tris-HCl, 20 mM acetic acid, 12.5 mM $MgCl_2$, 1 mM DTT, 1 µM $ZnCl_2$, 0.02% Tween20, 200 nM BSA, 100 nM calf thymus DNA and 200 mM NaCl at ambient temperature.

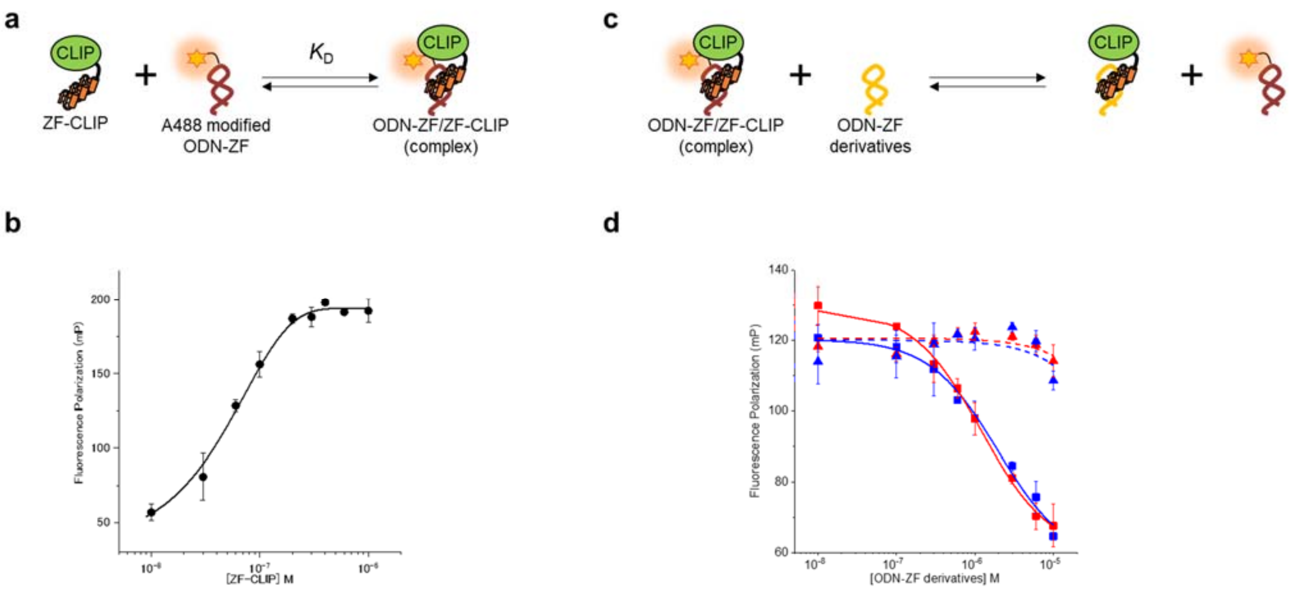

Figure S8 Fluorescence polarization analyses for the binding of ZF-CLIP to fluorophore modified ODN. (a-b) Titration of ZF-CLIP with the fluorophore modified ODN-ZF (A488-ODN-ZF) (5 nM) in the presence of 200 mM NaCl. (c-d) Competitive titration of ODN-ZF derivatives (ODN-ZF(G/T)-BC, ODN-ZF(G/C)-BC, ODN-ZF(GG/TC)-BC and ODN-ZF(GC/CT)-BC) (0 to 10 μM) toward the complex of ZF-CLIP (5 nM) and A488-ODN-ZF (40 nM). Reaction was conducted in a buffer (pH 8.0) containing 40 mM Tris-HCl, 20 mM acetic acid, 12.5 mM $MgCl_2$, 100 μM 2-mercaptoethanol, 1 μM $ZnCl_2$, 200 nM BSA, 0.002% Tween20 and 200 mM NaCl at 25 °C.

The fractional saturation θ for competition reaction can be represented as eq S3.[s10,11]

$$\theta = \{K_D + C\times K_D/K_C + P + D - [(K_D + C\times K_D/K_C + P + D)^2 - 4\times D\times P]^{1/2}\}/2D \qquad \text{(S3)}$$

P, D, and C represent the total concentrations of ZF-CLIP, A488-ODN-ZF and competitor ODN (ODN-ZF derivatives), respectively. K_D represents dissociation constant of ZF-CLIP and A488-ODN-ZF complex determined from Figure S8 (b) (56 nM), and K_C represents dissociation constant of the protein-competitor ODN complex, which were listed in Table S8.

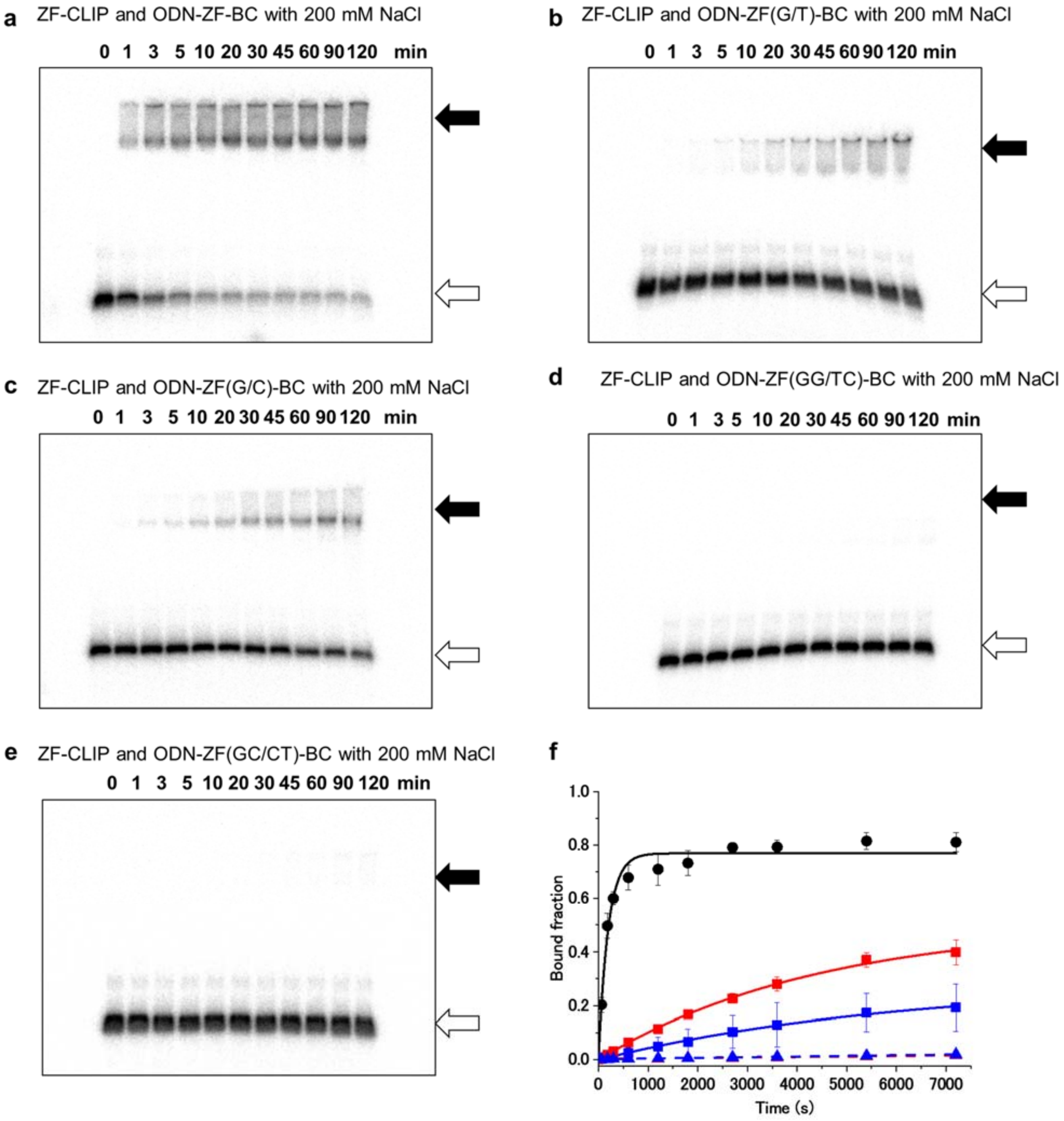

Figure S9 (a-e) Autoradiogram show denaturing gel electrophoretic analyses of the cross-linking reactions of 5'-^{32}P-end-labeled (a) ODN-ZF-BC, (b) ODN-ZF(G/T)-BC, (c) ODN-ZF(G/C)-BC, (d) ODN-ZF(GG/TC)-BC and (e) ODN-ZF(GC/CT)-BC with 100 nM ZF-CLIP. Open arrows and filled arrows denote free ODN and ZF-CLIP bound ODN, respectively. (f) Time-course plots for the crosslinking reaction of ODN-ZF-BC (black filled circle), ODN-ZF(G/T)-BC (blue filled square), ODN-ZF(G/C)-BC (red filled square), ODN-ZF(GG/TC)-BC (blue filled triangle) and ODN-ZF(GC/CT)-BC (red filled triangle) with ZF-CLIP to obtain the rate constants ($\boldsymbol{k}_{\mathbf{app}}$), which were listed in Table S9. Reaction was conducted in a buffer (pH 8.0) containing 40 mM Tris-HCl, 20 mM acetic acid, 12.5 mM $MgCl_2$, 100 µM 2-mercaptoethanol, 1 µM $ZnCl_2$, 0.02% Tween20, 200 nM BSA, 100 nM calf thymus DNA and 200 mM NaCl at ambient temperature.

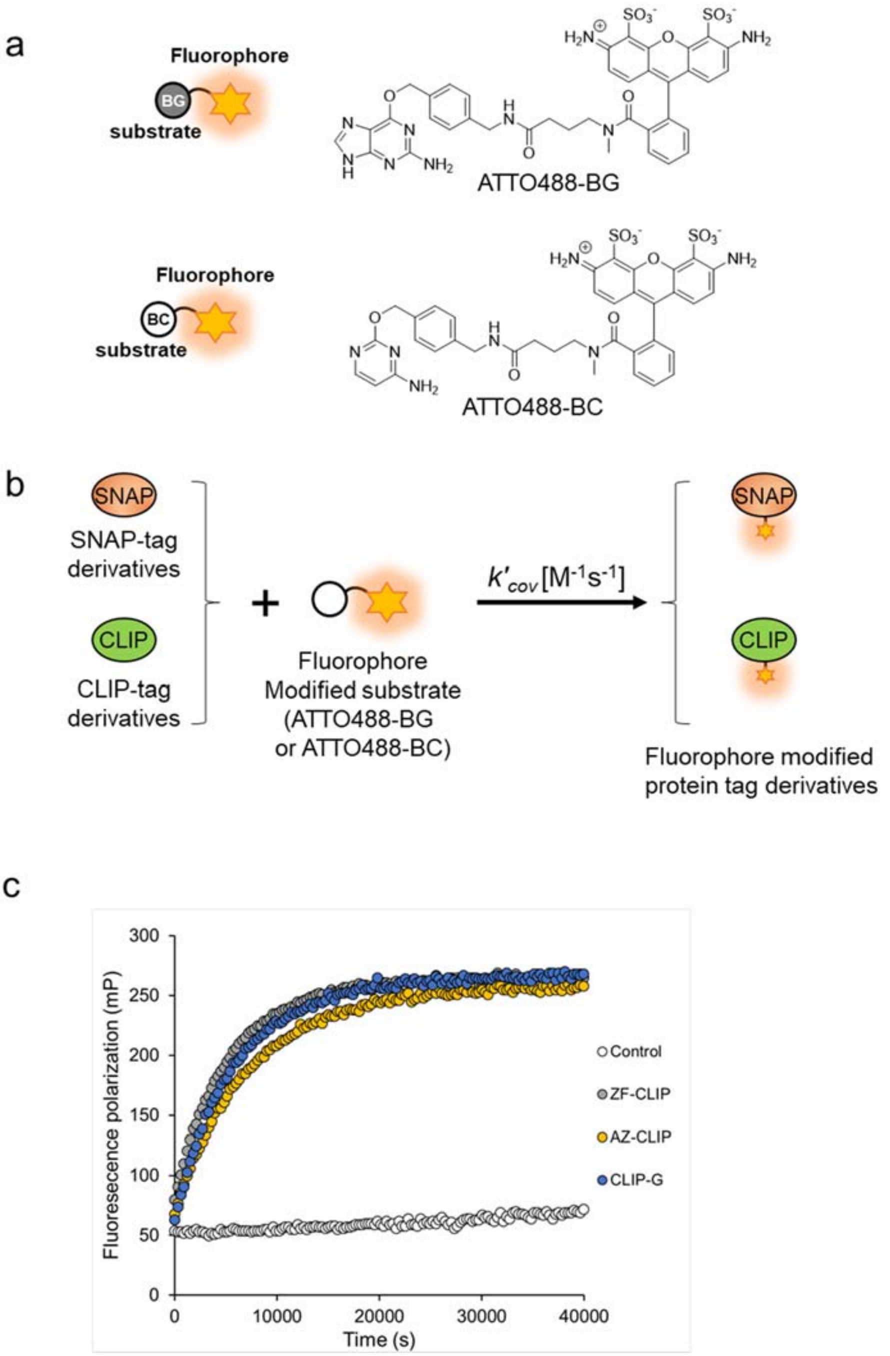

Figure S10 (a) Chemical structures of ATTO488 modified BG (ATTO488-BG) and ATTO488 modified BC (ATTO488-BC) (b) A scheme illustrates covalent bond formation between CLIP-tag (or SNAP-tag) derivatives with fluorophore modified substrates (ATTO488-BG or ATTO488-BC). (c) Time course changes of fluorescence polarization (mP) during the covalent bond formation between CLIP derivatives (2 μM) and ATTO488-BC (10 nM). Reaction was conducted in a buffer (pH 8.0) containing 40 mM Tris-HCl, 20 mM acetic acid, 12.5 mM $MgCl_2$, 1 mM DTT, 1 μM $ZnCl_2$, 200 nM BSA and 100 nM calf thymus DNA at ambient temperature. The estimated values are shown in Table S10 and S11.

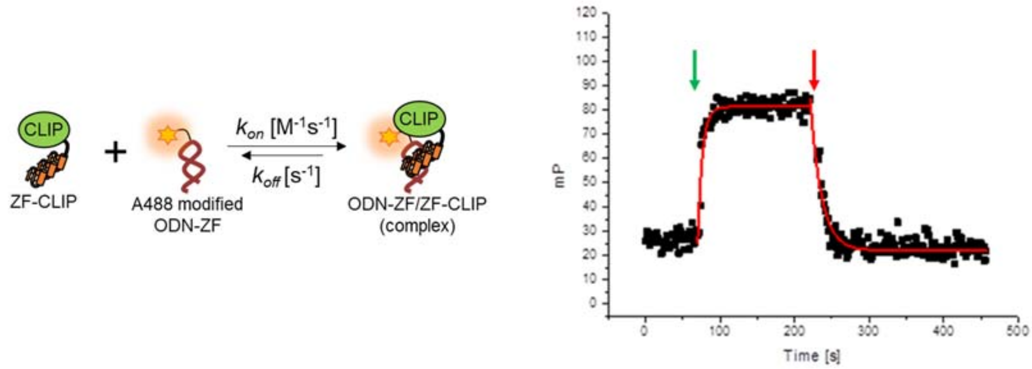

Figure S11 Fluorescence polarization analyses for the binding of ZF-CLIP to fluorophore modified ODN-ZF. Reaction was conducted in a buffer (pH 8.0) containing 40 mM Tris-HCl, 20 mM acetic acid, 12.5 mM $MgCl_2$, 1 mM DTT, 1 μM $ZnCl_2$, 100 nM calf thymus DNA and 0.02% Tween20 at 25 °C. Green arrow indicates the addition of 2 nM modular adaptor to the a solution containing 0.1 nM Alexa488 modified ODN-ZF (Table S1), and red arrow indicates the addition of 2 μM unlabelled ODN-ZF as the competitor for alexa488 modified ODN. The complex formation process was analysed with addition of various concentrations of modular adaptor (20 nM ~ 100 nM) to 1 nM alexa488 modified ODN. The kinetic parameter k_{obs} (s^{-1}) was obtained by fitting to a reaction model assuming the first-order kinetics (eq S1), and then k_{on} ($M^{-1}s^{-1}$) and k_{off} (s^{-1}) was determined by following eq S2. The dissociation process was also monitored by addition of an excess amount of unlabeled ODN (2 μM) to the reaction mixture. The estimated values are shown in Table S12.

$$P = (P_{max} - P_0)(1 - e^{-k_{obs}t}) + P_0 \qquad \text{(S1)}$$

P, P_{max} and P_0 represent the fluorescence polarization at the certain time, the maximum fluorescence polarization, and the fluorescence polarization in the absence of MA, respectively.

$$k_{obs} = k_{on} \times [MA] + k_{off} \qquad \text{(S2)}$$

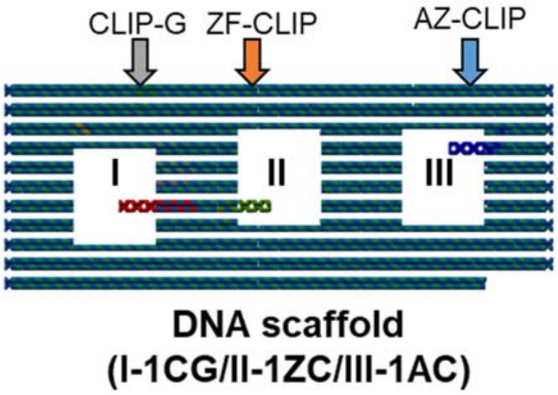

Figure S12 An illustration of DNA scaffold used in this study. 3-well DNA scaffold contains three single binding sites at cavity I, II, and III for CLIP-G, ZF-CLIP and AZ-CLIP, respectively.

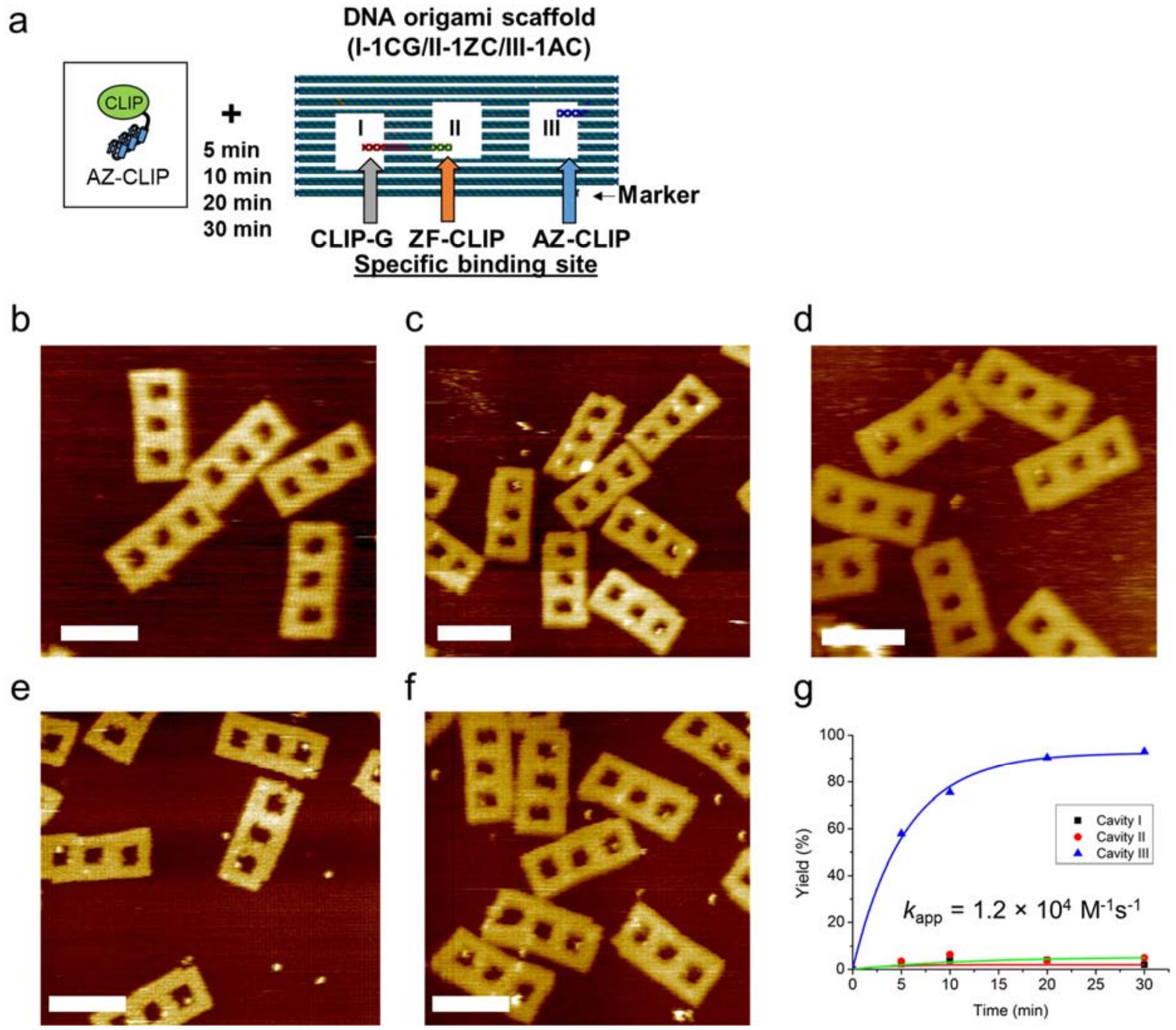

Figure S13 Reaction of AZ-CLIP to the matched binding site (cavity III) and the unmatched binding site (cavity I and cavity II) on 3-well DNA scaffold (I-1CG/II-1ZC/III-1AC). (a) An illustration for the assembly reaction of AZ-CLIP on DNA scaffold I-1CG/II-1ZC/III-1AC**.** (b-f) 5 nM DNA scaffold (I-1CG/II-1ZC/III-1AC) was incubated with 250 nM AZ-CLIP. An aliquot of the reaction mixture was purified by size-exclusion chromatography after (b) 0 min, (c) 5 min, (d) 10 min, (e) 20 min and (f) 30 min incubation, then analysed by AFM. The reaction was carried out in a buffer (pH 8.0) containing 40 mM Tris-HCl, 20 mM acetic acid, 12.5 mM $MgCl_2$, 1 mM DTT, 1 µM $ZnCl_2$, 0.02% Tween20, and 200 mM NaCl. (g) The assembly yield of AZ-CLIP at each cavity was estimated by counting the number of cavities occupied by AZ-CLIP. The estimated k_{app} for the reaction of AZ-CLIP at matched binding site on DNA origami scaffold was 1.2 × 10^4 $M^{-1}s^{-1}$.

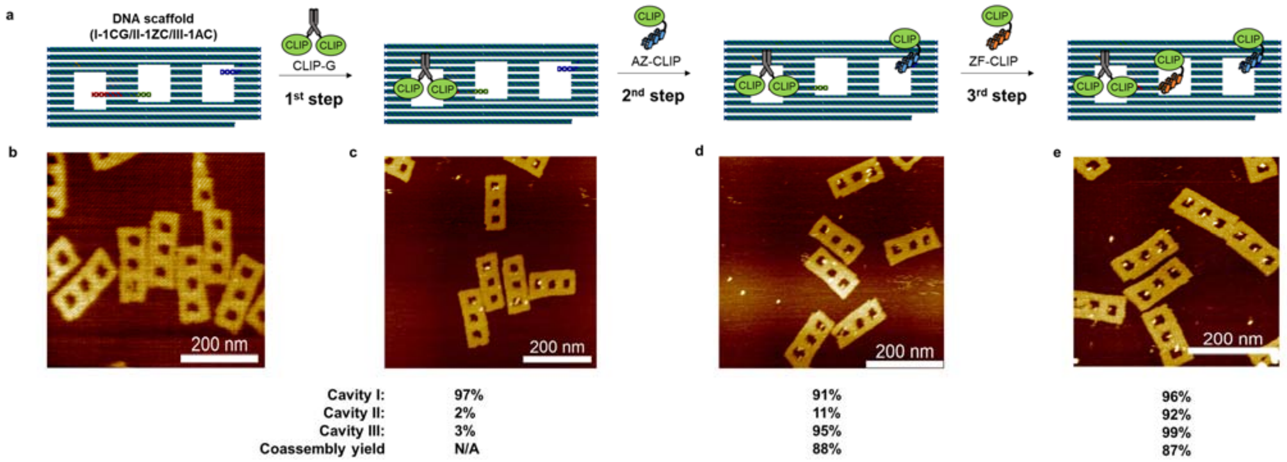

Figure S14 (a) An illustration for sequential assembly of three types of modular adaptors on the DNA scaffold (I-1CG/II-1ZC/III-1AC). (b−e) AFM images of the DNA scaffold reacted with the modular adaptors. The DNA scaffold (5 nM) was incubated (c) with CLIP-G (250 nM) for 20 min at ambient temperature (1st step), then (d) with the AZ-CLIP (250 nM) for additional 20 min (2nd step), and finally (e) with ZF-CLIP (250 nM) for further 5 min (3rd step). The reactions were carried out in a buffer (pH 8.0) containing 40 mM Tris-HCl, 20 mM acetic acid, 12.5 mM $MgCl_2$, 200 mM NaCl, 1 mM DTT, 1 µM $ZnCl_2$, and 0.02% Tween20. At each step, an aliquot of the reaction mixture was purified by size-exclusion chromatography, then analysed by AFM. Modification yield for each cavity and the coassembly yields after 2nd and 3rd steps were estimated by counting the number of cavities occupied by the modular adaptors.

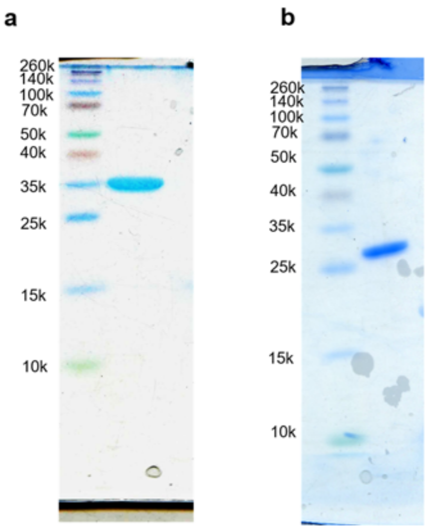

Figure S15 SDS-PAGE analyses of purified modular adaptors. (a) ZF-CLIP, (b) CLIP-G. Amino acid sequences and molecular weights of these proteins are shown in Table S15.

References

S1 B. A. Barshop, R. F. Wrenn, C. Frieden, *Anal. Biochem.* 1983, **130**, 145.

S2 C. T. Zimmerle, C. Frieden, *Biochem. J.* 1989, **258**, 381.

S3 Y. H. Tsai, S. Essig, J. R. James, K. Lang, J. W. Chin, *Nat. Chem.* 2015, **7**, 554.

S4 M. Geertz, D. Shore, S. J. Maerkl, *Proc. Natl. Acad. Sci. U. S. A.* 2012, **109**, 16540.

S5 A. Gautier, A. Juillerat, C. Heinis, I. R. Corrêa Jr., M. Kinder mann, F. Beaufils, K. Johnsson, *Chem. Biol.* 2008, **15**, 128.

S6 O. G. Berg, R. B. Winter, P. H. von Hippel, *Biochemistry* 1981, **20**, 6929.

S7 J. S. Kim, C. O. Pabo, *Proc. Natl. Acad. Sci. U. S. A.* 1998, **95**, 2812.

S8 S. E. Halford, J. F. Marko, *Nucleic Acids Res.* 2004, **32**, 3040.

S9 S. E. Halford, *Biochem. Soc. Trans.* 2009, **37**, 343.

S10 P. Thordarson, *Chem. Soc. Rev.* 2011, **40**, 1305.

S11 T. Morii, Y. Saimei, M. Okagami, K. Makino, Y. Sugiura, *J. Am. Chem. Soc.* 1997, **119**, 3649.
